# Selective Tracking of Charge Carrier Dynamics in CuInS_2_ Quantum Dots

**DOI:** 10.1021/acsnano.4c18469

**Published:** 2025-06-12

**Authors:** Andrés Burgos-Caminal, Brener R. C. Vale, André F. V. Fonseca, Elisa P. P. Collet, Juan F. Hidalgo, Lázaro García, Luke Watson, Olivia Borrell-Grueiro, María E. Corrales, Tae-Kyu Choi, Tetsuo Katayama, Dongxiao Fan, Víctor Vega-Mayoral, Saül Garcia-Orrit, Shunsuke Nozawa, Thomas J. Penfold, Juan Cabanillas-González, Shin-Ichi Adachi, Luis Bañares, Ana Flávia Nogueira, Lázaro A. Padilha, Marco Antônio Schiavon, Wojciech Gawelda

**Affiliations:** † Madrid Institute for Advanced Studies in Nanoscience, 202533 IMDEA Nanociencia, Ciudad Universitaria de Cantoblanco, Calle Faraday 9, Madrid 28049, Spain; ‡ Departamento de Química, Universidad Autónoma de Madrid, Ciudad Universitaria de Cantoblanco, Calle Francisco Tomás y Valiente 7, Madrid 28049, Spain; § Instituto de Física Gleb Wataghin, 28132 Universidade Estadual de Campinas - UNICAMP, Campinas 13083-852, São Paulo, Brazil; ∥ Grupo de Pesquisa Química de Materiais, Departamento de Ciências Naturais, Universidade Federal de São João Del-Rei, São João Del-Rei 36307-352, Minas Gerais, Brazil; ⊥ Laboratório de Nanotecnologia e Energia Solar, Instituto de Química, 28132 Universidade Estadual de Campinas - UNICAMP, Campinas 13083-852, São Paulo, Brazil; # Chemistry-School of Natural and Environmental Sciences, 5994 Newcastle University, Newcastle upon Tyne NE1 7RU, U.K.; ∇ Departamento de Química Física and Center for Ultrafast Lasers, Facultad de Ciencias Químicas, Universidad Complutense de Madrid, Madrid 28040, Spain; ○ Departamento de Química Física Aplicada, 16722 Universidad Autónoma de Madrid, Ciudad Universitaria de Cantoblanco, Calle Francisco Tomás y Valiente 7, Madrid 28049, Spain; ◆ XFEL Division, Pohang Accelerator Laboratory, POSTECH, Pohang 37673, Gyeongbuk, Republic of Korea; ¶ 133704 Japan Synchrotron Radiation Research Institute, Kouto 1-1-1, Sayo 679-5198, Hyogo, Japan; & Institute of Materials Structure Science, High Energy Accelerator Research Organization (KEK), 1-1 Oho, Tsukuba 305-0801, Ibaraki, Japan; ● Faculty of Physics, Adam Mickiewicz University, ul. Uniwersytetu Poznańskiego 2, 61-614 Poznan, Poland

**Keywords:** CuInS_2_ quantum dots, XFEL, X-ray absorption, transient absorption, charge carrier dynamics, hole trapping

## Abstract

CuInS_2_ quantum dots have been studied in a broad range of applications, but despite this, the fine details of their charge carrier dynamics remain a subject of intense debate. Two of the most relevant points of discussion are the hole dynamics and the influence of Cu:In synthesis stoichiometry. It has been proposed that Cu-deficiency leads to the formation of Cu^2+^, affecting the localization of holes into Cu defects. Importantly, it is precisely these confined hole states that are used to explain the interesting photoluminescence properties of CuInS_2_ quantum dots. We use static X-ray spectroscopy to show no evidence for a measurable amount of native Cu^2+^ states in Cu-deficient samples (above 20%). Instead, the improved properties of these samples are explained by an increase of crystallinity, reducing the concentration of mid-gap states. Furthermore, to understand the charge carrier dynamics, herein, we employ ultrafast optical transient absorption and fluorescence up-conversion spectroscopies in combination with ultrafast X-ray absorption spectroscopy using a hard X-ray free electron laser. We demonstrate that in nonpassivated samples, holes are transferred from Cu atoms on subpicosecond time scales. Finally, we observe that Cu-deficient samples are more robust against photothermal effects at higher laser fluences. This is not the case for the Cu-rich sample, where heating effects on the structure are directly observed.

## Graphical abstract

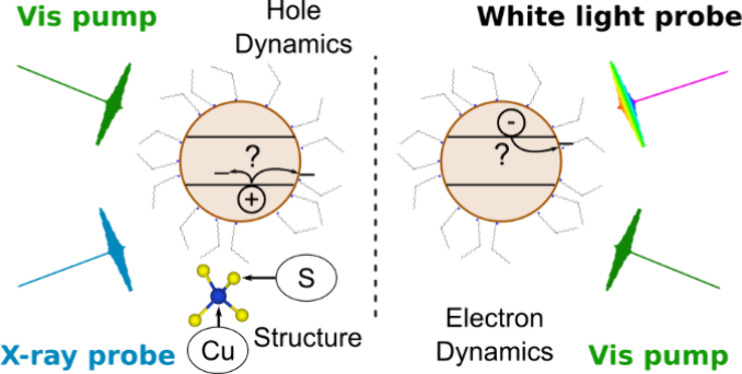

CuInS_2_ quantum dots (CIS QDs) have found applications across diverse fields including photovoltaics,^1–4^ LEDs,^5–7^ solar concentrators,^8–10^ and biological labeling.^11,12^ This diversity of applications comes from their unique optical properties such as high molar absorption coefficient (>10^4^ L mol^–1^ cm^–1^), large Stokes shift (from 200 to 400 meV), which makes them free of reabsorption effects, long charge carrier recombination times (more than hundreds of nanoseconds), and fluorescence that can be tuned by the diameter of the QD.^13^ Furthermore, they are free of the highly toxic Cd or Pb, typically used in other QDs. Yet, CIS QDs usually have a low photoluminescence quantum yield (PLQY) due to a high density of defects. To overcome this limitation, approaches involving core–shell structures have been used, achieving PLQY values as high as 50%.^14–16^

The most accepted model for the photoluminescence (PL) mechanism of CIS QDs is that it derives from a recombination of a delocalized electron in the conduction band with a confined hole state (CHS).^14,17–20^ This CHS is related to Cu defects, which are about 100 meV above the material’s valence band. Ultrafast spectroscopy measurements suggest that after the photoexcitation, hole trapping occurs on a time scale spanning from hundreds of femtoseconds to a few picoseconds.^14,17,21^ This is followed by two competing processes: electron trapping at sub-band gap trap states located mainly at the QD surface,^22,23^ and radiative recombination of the electron with the CHS.^14,17,21^

The oxidation state of the Cu-related defects, acting as localization centers for the CHS, also plays a critical role in controlling the optical properties of CIS QDs. With respect to absorption, the current understanding is that defects of Cu^+^ have an active absorption fingerprint due to excitation from a 3d^10^ state to the conduction band of the material, which would be responsible for the broad absorption tail below the bandgap.^21,24^ Contrarily, defects related to Cu^2+^, already containing a CHS, do not affect the absorption spectrum with such a tail because a transition from a 3d^9^ electronic configuration would require energy above the bandgap. Furthermore, the recent literature explains the emission mechanism from the CHS in the following way: after photoexcitation in the material’s bandgap, a fast hole transfer from the valence band to the Cu^+^ defects must occur, forming the CHS. Alternatively, the emission from native Cu^2+^ defects promptly occurs due to the already present vacancy in the 3d^9^ electronic configuration; however, another hole trapping mechanism, not related to Cu, needs to quickly remove the photogenerated hole from the valence band to account for the observed high PLQY. Otherwise, an Auger recombination involving the two holes (valence band and d^9^ vacant orbital) and the electron in the conduction band would suppress the emission.^21,22^

According to recent reports, an efficient way to control the defects related to Cu is by varying the QD stoichiometry during the synthesis. Based on charge neutralization arguments, in stoichiometric samples (Cu/In = 1), most of the defects are cation antisite, where Cu^+^ is located in a site of In^3+^ with a double negative charge and an In^3+^ is localized in a Cu^+^ site with a double positive charge. Following the Kröger–Vink notation of crystallographic defects, this is represented as Cu_In_″–In_Cu_^··^. Moreover, Cu deficiency leads to Cu vacancy (v_Cu_′), therefore, charge neutralization creates Cu^2+^ (Cu_Cu_^·^).^13,21,22^ In addition, CIS QDs have low PLQY due to surface trap states and, to increase this parameter, the QDs are shelled with a wide bandgap semiconductor material. One good candidate for this task is the insulator ZnS.^12,25,26^

Typically, the characterization of CIS QDs, and especially of their charge carrier dynamics, is done employing UV–vis and sometimes NIR spectroscopy techniques.^13,17,22,27^ Some studies have included the use of X-ray absorption spectroscopy (XAS),^28^ including time-resolved XAS (TR-XAS).^29^ However, no study before has been able to take advantage of TR-XAS with femtosecond time resolution, thanks to the recent development of X-ray free electron lasers (XFELs). Measuring physicochemical properties in colloidal dispersion is advantageous because procedures for obtaining nanocrystals (NCs) as a solid can affect their properties. Based on that, UV–vis absorption and PL measurements have been the most used techniques to characterize different processes due to their simplicity in their application to colloids. However, other more specific approaches that allow the characterization of NCs in a colloidal dispersion are highly desirable. Here, we used steady-state XAS and femtosecond TR-XAS to investigate colloidal CIS QDs. X-ray spectroscopy has a considerable advantage with respect to UV–vis. It is sensitive to structural and oxidation state changes, and it has element specificity. This means that we can probe the oxidation state changes in Cu upon the formation of a valence band hole, or the structural changes between samples.^30^ In addition, using XAS techniques enables us to measure structural properties directly in a colloidal dispersion, unlike other X-ray or electron techniques such as X-ray powder diffraction, X-ray photoelectron spectroscopy, and transmission electron microscopy. Here, we have employed, for the first time, femtosecond hard X-ray pulses from the SACLA XFEL (Hyogo, Japan) to study the ultrafast dynamics of CIS QDs. We have studied three different samples to understand how stoichiometry and the presence of a passivation layer affects the optical properties of the material. Our main findings are concentrated in understanding the role of Cu:In stoichiometry and surface passivation on the behavior of both charge carriers. We probed the effects on the steady-state UV–vis and X-ray spectra, and the corresponding ultrafast dynamics of CIS QDs, showing results that contradict some of the current understanding of the photophysics and the compositional effects, prompting further investigation into these systems.

## Results and Discussion

We investigated three different CIS QD samples to understand the effects of synthesis stoichiometry and surface passivation on the structure and charge carrier dynamics: (a) stoichiometric (100%) bare CIS QDs, (b) Cu-deficient (20%) bare CIS QDs, and (c) a passivated Cu-deficient sample, doped with Zn and submitted to a post-treatment with ZnCl_2_ at 200 °C. This treatment gives the QDs a core–shell structure, passivating the surface and minimizing nonradiative recombination. Therefore, this last sample allows us to better observe the hole behavior without the interference of other trap-assisted processes at the surface. The elemental analysis carried out with X-ray fluorescence (Table S1) shows that we are studying extreme cases of stoichiometry, where sample (a) presents an excess of Cu, and thus is Cu-rich, while (b) and (c) are Cu-deficient. Hereon, we will refer to the samples by their formula in Table S1: (a) CuIn_0.4_S_*x*_, (b) Cu_0.3_InS_*x*_, and (c) Cu_0.2_InS_*x*_/ZnS.

The detailed synthesis method, based on previously published work,^14,31^ can be found in the Methods, yielding CIS QDs expected to have a chalcopyrite structure^32–34^ (Figure 1A), although it may be difficult to distinguish from a face-centered cubic structure.^31^ X-ray diffraction (XRD, Figure S1), reveals a good agreement with chalcopyrite for Cu-deficient samples, while CuIn_0.4_S_*x*_ may have a small contribution from wurtzite, pointing to a larger disorder with this stoichiometry. Size analysis with electron microscopy (Figure S2) shows a larger size for CuIn_0.4_S_*x*_ (5.3 ± 0.6 nm) compared to Cu_0.3_InS_*x*_ (3.7 ± 0.7) or Cu_0.2_InS_*x*_/ZnS (3.3 ± 0.5).

**Figure 1:**
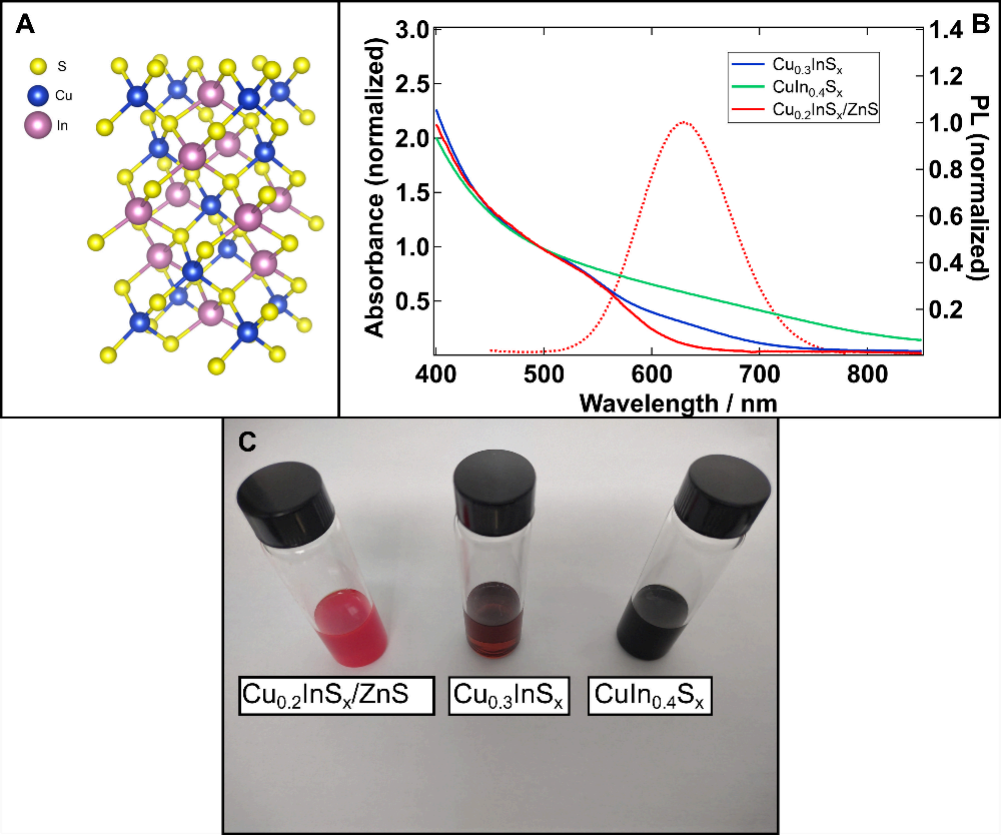
(A) Crystal structure of chalcopyrite CuInS_2_.^32,35^ (B) Steady-state absorption spectra of the three samples under study (solid lines normalized at 500 nm), as well as the PL signal of Cu_0.2_InS_*x*_/ZnS, the only highly emissive sample (dashed line). (C) Three samples under study: Cu_0.2_InS_*x*_/ZnS, Cu_0.3_InS_*x*_, and CuIn_0.4_S_*x*_.

The UV–vis absorption and PL are shown in Figure 1B. We can already identify prominent sub-bandgap transitions below the excitonic absorption energy at 550 nm in the nonpassivated samples CuIn_0.4_S_*x*_ and Cu_0.3_InS_*x*_, corresponding to defect states. Furthermore, this is considerably larger in CuIn_0.4_S_*x*_ and the excitonic band is not distinguishable, pointing to a higher degree of disorder. Last, only the well-passivated Cu_0.2_InS_*x*_/ZnS sample shows a considerable PL signal (Figure S3), which correlates with the lower density of trap states producing only a small Urbach tail and having a PLQY of 29.8 ± 0.3%. These changes in absorption and PL give clear differences in the appearance of the samples (Figure 1C).

### Cu K-edge X-ray Absorption Spectroscopy

We measured the Cu K-edge X-ray absorption spectra of our three samples at the CLÆSS beamline of the ALBA synchrotron, with the goal of studying the effect of stoichiometry and finding evidence for the presence of Cu^2+^ in Cu-deficient samples (Figure 2). We obtained both the X-ray absorption near-edge structure (XANES) and the extended X-ray absorption fine structure (EXAFS) spectra.

**Figure 2:**
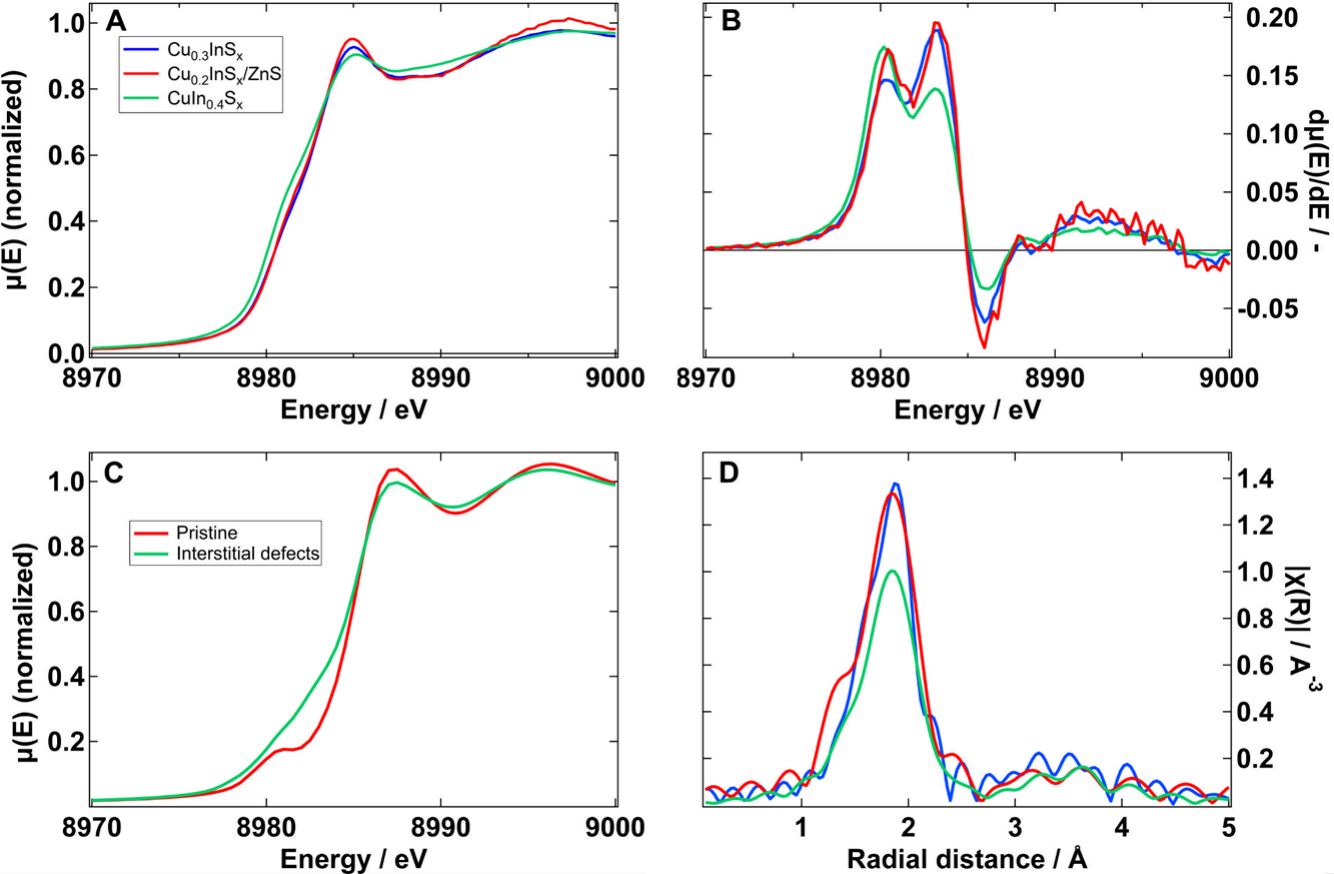
(A) XANES spectra of the three samples under study. (B) First derivative of the XANES spectra lacking any evidence of a prepeak around 8978 eV. (C) Simulations of XANES spectra including interstitial defects to simulate disorder. (D) EXAFS of the corresponding samples. The main differences between the Cu-rich and Cu-deficient samples are a broadening of the XANES peaks, and a lowering of the EXAFS one. This is attributed to disorder in the sample. A similar effect is seen in the simulations. A, B, and D share the same color code to represent the samples.

At first glance, in Figure 2A, it may appear that the K-edge of CuIn_0.4_S_*x*_ is shifted to lower energies compared to the others. However, as it will be explained below, this is not true. In addition, the Cu-rich sample shows a suppressed white line at 8985 eV compared to the Cu-deficient Cu_0.3_InS_*x*_ and Cu_0.2_InS_*x*_/ZnS. This band is ascribed to a 1s → 4p transition, and its energy position and intensities are sensitive to the local structure of the Cu^+^ species.^36^ Simulations reported by van der Stam et al.^28^ show that a more intense transition is associated with an increase of positive charge on the absorbing atom and thus implies an increase in its average oxidation state. Such behavior was studied experimentally by applying electrochemical potentials, but the changes in the XANES spectrum were not conclusive.^28^ Hu et al. have demonstrated that this peak is instead enhanced with increasing NCs size.^29^ Furthermore, this enhancement is attributed to tetrahedrally coordinated Cu^+^ and increasing uniformity, while a smoother feature is characteristic of a triangular coordination and disorder.^29,37^

Therefore, the changes observed in our data can either be due to a decrease in the disorder of the tetrahedral structure or to a change in the oxidation state.

A typical signature that reveals the presence of Cu^2+^ is the presence of a pre-edge peak in the 8975–8980 eV region due to a quadrupole-allowed 1s → 3d transition.^37,38^ An example can be seen in a reference spectrum in Figure S4. However, all three CIS QDs show no such feature in their XANES spectra or their derivative (Figure 2A,B). Thus, we exclude the possibility of a considerable proportion of Cu^2+^ states, since we do not detect this transition. If Cu^2+^ is present, its density is below the detection limit which in this measurement lies at ca. 20% of Cu^2+^ (Figure S5). Furthermore, no consistent shift is observed in the different features, compared to the Cu^+^ reference sample (Figure S6). Indeed, the postedge peak at 8995 eV, ascribed to another 1s → 4p transition of Cu^+^, is intensified while moving from Cu-rich to Cu-deficient samples, but does not shift. At the same time, a mixture of Cu^2+^ and Cu^+^ states in the Cu-deficient samples would result in broader peaks due to this shift, while we observe the opposite behavior. Moreover, spectroelectrochemical measurements show that the PLQY of QDs decreases upon applying an oxidative potential through the formation of Cu^2+^.^28^ However, our PL data shows the opposite behavior: the PL is enhanced for Cu-deficient samples, which are supposed to have higher density of Cu^2+^.^22^ In addition, the same behavior has been observed before for Cu_2_ZnSnS_4_ (CZTS) films, by Turnbull et al.^37^ In that study, the difference between films before and after annealing is the same that we have in Figure 2A between CuIn_0.4_S_*x*_ and Cu_0.2_InS_*x*_/ZnS, showing a direct link between these changes and crystallinity. Note that CZTS has a very similar structure to CIS where Cu is tetrahedrally coordinated to 4 S atoms. It is possible that the shoulder on the rising edge at 8981 eV is characteristic of a lower coordination, explaining the enhancement in CuIn_0.4_S_*x*_.^39^ Therefore, Cu K-edge XANES measurements suggest that Cu-deficient samples favor the tetrahedral coordination of Cu–S and a decrease in disorder. This conclusion is supported by other studies in the literature.^29,37,40^ Furthermore, we can simulate the same behavior of the XANES spectra by introducing interstitial defects as a representation of disorder (Figure 2C).

In Figure 2D we present the Cu K-edge EXAFS. We note that the main feature centered at ∼1.9 Å has higher intensity for the Cu-deficient Cu_0.3_InS_*x*_ and Cu_0.2_InS_*x*_/ZnS. This is associated with either an increase in the coordination number or a decrease in disorder around the metal center.^29^

We performed a FEFF6 fitting analysis of the Cu K-edge EXAFS data with the Demeter package,^32,41^ and we observed that the coordination number of Cu increases from 2 for the Cu-rich sample to 3 for the Cu-deficient ones, pointing to a larger disorder and defects in the former. Certainly, the ideal value is expected to be higher for the bulk material, since each Cu atom is surrounded by 4 S atoms. However, coordination numbers between 2 and 3 agree with the data from the literature for CIS QDs.^29,37^ The reduced coordination number is due to the large proportion of surface atoms compared to the total volume. Moreover, the fitted bond length of Cu–S increases slightly while moving from Cu-rich to Cu-deficient samples. These results are summarized in Table S3, and the corresponding fit curves are shown in Figure S7. In short, Cu K-edge EXAFS results confirm that the Cu deficiency increases the coordination number and decreases disorder. It is worth noting that these results can also be explained by a transition from an In-dominated surface in Cu-deficient Cu_0.3_InS_*x*_ and Cu_0.2_InS_*x*_/ZnS, to a Cu-dominated one for the Cu-rich CuIn_0.4_S_*x*_. This would induce a general decrease in Cu coordination and may explain the introduction of defect states that serve as recombination centers.

### Transient Absorption Spectroscopy

We now turn to time-resolved measurements to study the effect of composition on the charge carrier dynamics. First, we analyze ultrafast optical transient absorption (OTAS) measurements, which are mainly sensitive to the population of charge carriers, in the form of a strong ground state bleach (GSB) and weak excited state absorption (ESA). This is seen as a negative Δ*A* signal in a broad region, resonant with the excitonic absorption of the sample, and a positive signal, respectively.^42,43^

Due to the much larger effective mass of holes in these systems, OTAS is mainly sensitive to the evolution of electrons.^13,44^

In Figure 3 we show the OTAS results for our three distinct samples, Cu_0.2_InS_*x*_/ZnS, CuIn_0.4_S_*x*_, and Cu_0.3_InS_*x*_. We can observe that the probed dynamics at different wavelengths of Cu_0.2_InS_*x*_/ZnS are almost flat in a time window of 4 ps, but for CuIn_0.4_S_*x*_ the GSB decays much faster. Alternatively, the behavior of Cu_0.3_InS_*x*_ lies in between the other two. We apply a global fit of a three- or four-exponential model to characterize the time evolution of the transient absorption signal. This analysis results in the corresponding fit lines on the left plots and the decay-associated spectra (DAS) in the right plots. The DAS shows the wavelength dependence of the amplitude of each of the fitted lifetimes. Thus, it allows us to interpret spectral differences between decays. A representation of the different charge carrier evolution pathways is shown in Scheme 1 and referenced throughout the text with numbers **1** to **6**. We observe a blueshift in the main GSB signal when going progressively from CuIn_0.4_S_*x*_ to Cu_0.3_InS_*x*_ and to Cu_0.2_InS_*x*_/ZnS owing to the effects of Zn-doping and stoichiometry, similar to the effects on steady-state absorption (Figure 1B). Interestingly, the shortest decay shows a different DAS from the other ones. It is seen as an early decay of the blue side of the GSB on Cu_0.2_InS_*x*_/ZnS and as a shift of the GSB in CuIn_0.4_S_*x*_ and Cu_0.3_InS_*x*_. Previous reports have attributed these changes in the GSB to hole trapping into the CHS (**1**).^14,45,46^ This may be true for the relatively small effect of τ_1_ on the Cu_0.2_InS_*x*_/ZnS sample. However, the large effects seen on the bare samples must be related to fast charge trapping at states below the excitonic band (**2** and **3**), probably related to the surface. Similar two-band structures have been observed with different proportions as the stoichiometry is changed.^23,43,47^ Jara et al. showed a similar behavior to what is observed here for CuIn_0.4_S_*x*_ and Cu_0.3_InS_*x*_ and assigned it to Cu states above the valence band.^47^ However, in this case, the lack of the structure in the PL and the disappearance in Cu_0.2_InS_*x*_/ZnS with surface passivation points to surface states. In some aspects, our results are distinguishably different from previously reported data for Cu-deficient CIS QDs.^23,43,47^ Indeed, we observe differences in PLQY, UV–vis absorption spectrum and the underlying transitions, which may involve the excited states, which are not fully relaxed on fs time scales measured by OTAS. Altogether the observed static and dynamic properties point to a difference in general properties compared with some of the earlier reported studies. Such differences may certainly stem from the difference in the synthetic procedure and in the capping ligands employed. These should be considered when comparing our results with different CIS QDs.

**Figure 3:**
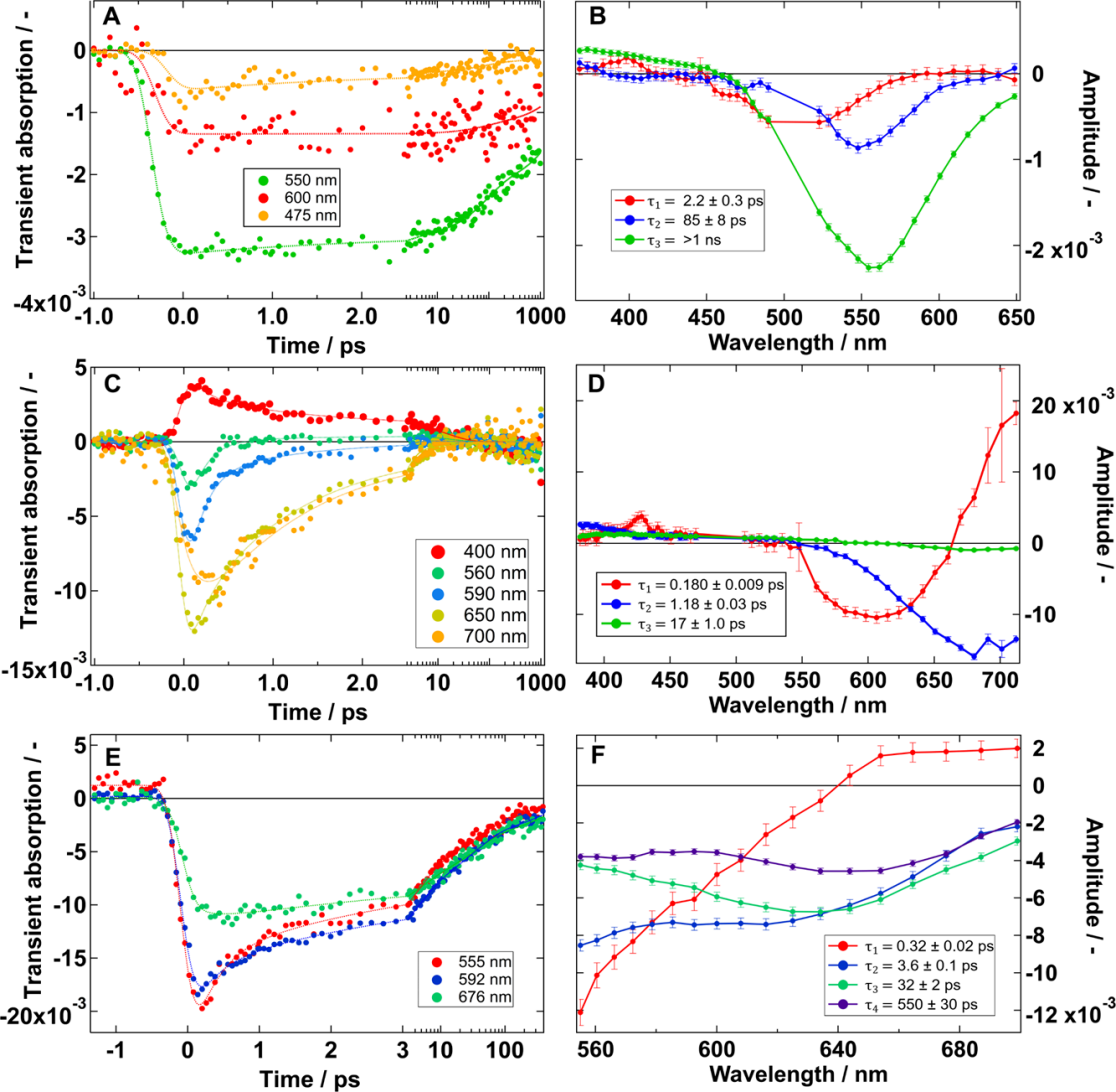
OTAS of Cu_0.2_InS_*x*_/ZnS (A, B), CuIn_0.4_S_*x*_ (C, D), and Cu_0.3_InS_*x*_ (E, F) pumped at 520 nm and 0.08, 0.6, and 1.0 mJ·cm^–2^, respectively. The left shows the time evolution at selected wavelengths while the right shows the decay-associated spectra obtained through a global fit to a multiexponential model. We used low fluence in Cu_0.2_InS_*x*_/ZnS to avoid multiexcitonic effects. In contrast, we used higher fluences for the other two samples because: (a) trapping dominates over multiexcitonic effects, as seen on the X-ray measurements below and Figure S9, and (b) it was needed to improve the signal-to-noise in these two systems.

**Scheme 1:**
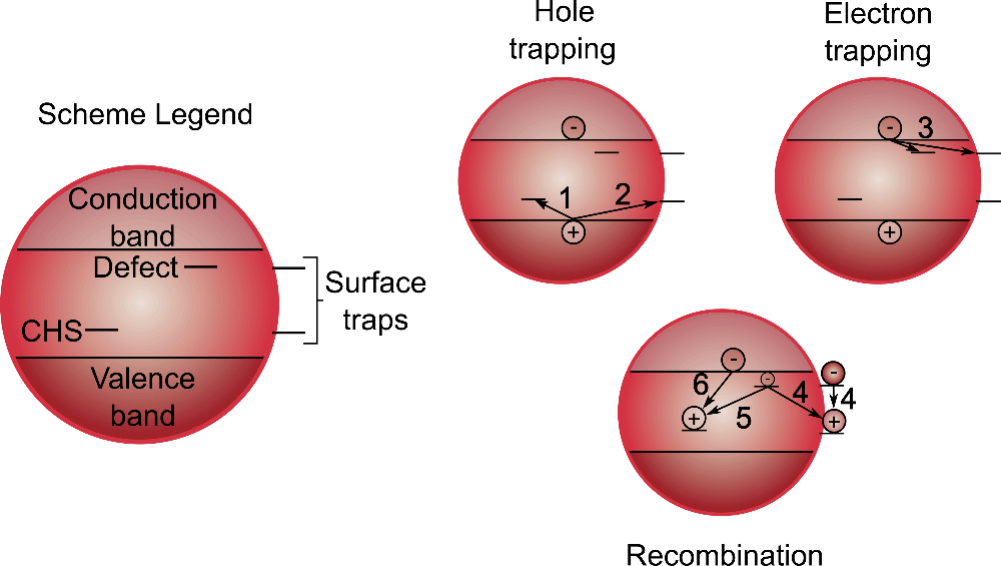
Representation of Charge Carrier Pathways^a^

a (1) CHS formation, (2) hole surface trapping, (3) electron trapping, (4) nonradiative recombination of trapped carriers, (5) nonradiative recombination with CHS, and (6) radiative recombination with CHS.

Cooling of charge carriers in the bands is not observed since we pump at the excitonic band with 520 nm. Later lifetimes correspond to the recombination of the trapped carriers (**4**). This explains the lack of PL in CuIn_0.4_S_*x*_ and Cu_0.3_InS_*x*_. In addition, it confirms that the tails of absorption below the excitonic peak in both samples correspond to abundant trap states related to (1) the bare surface (for both), and (2) the disorder in CuIn_0.4_S_*x*_. The results also showed that the Cu-deficient sample recovered much slower than the Cu-rich one due to the lack of the second source of traps from internal defects. In the case of the passivated sample, Cu_0.2_InS_*x*_/ZnS, we can ascribe the second (A2) and third (A3) time constants to electron trapping (**3**) and nonradiative recombination (**5**), in which trapped electrons recombine with the hole in the CHS. These lifetimes correspond to a portion of the QDs that are susceptible to trapping due to the lower presence of defects compared to the other samples.^14,17^ From time-resolved PL (Figure S14 and Table S4) measurements of the most passivated sample (Cu_0.2_InS_*x*_/ZnS), we can deduce a radiative recombination (**6**) lifetime of ca. 300 ns.

In agreement with the steady-state data, the Cu-deficient samples present lower electron traps and nonradiative rates (Table 1), confirming that those samples have much better optical properties than the Cu-rich one.

**Table 1:** Electron Trapping (**3**) and Nonradiative (**4**, **5**) Time Constants for the Samples Obtained from OTAS

sample	τ_electron trapping_/ps	τ_nonradiative_/ps
CuIn_0.4_S_*x*_	1.18 ± 0.03	17 ± 1.0
Cu_0.3_InS_*x*_	18^a^ ± 1	550 ± 30
Cu_0.2_InS_*x*_/ZnS	85 ± 8	>1000

a The electron trapping lifetime for Cu_0.3_InS_*x*_ has been taken as an average of the two middle constants since the amplitudes are similar.

### Time-Resolved X-ray Absorption Spectroscopy

In order to gather more insights into the behavior of holes, we employed TR-XAS by probing the Cu K-edge. We chose to probe there because Cu contributes to a large degree to the valence band states, and the CHS is also attributed to Cu defects. The data was acquired at the SACLA XFEL.

The left side of Figure 4 shows the steady-state and time-resolved XANES spectra at the Cu K-edge, and the right side shows the dynamics at specific energies. We can observe that the steady-state spectra, which in this case are obtained while the pumping laser at 520 nm is off, resemble closely the spectra obtained at the ALBA synchrotron (Figures 2A and S10). This confirms that we are working with equivalent samples. We can observe in Figure 4A,C that immediately after photoexcitation (1.2, 0.14–0.54, and 0.44 ps pump–probe delays, for Cu_0.2_InS_*x*_/ZnS, CuIn_0.4_S_*x*_, and Cu_0.3_InS_*x*_, respectively) the typical signature of Cu^2+^ is present as a photoinduced absorption at 8978 eV and a large negative band denoting a blue-shift of the edge.

**Figure 4:**
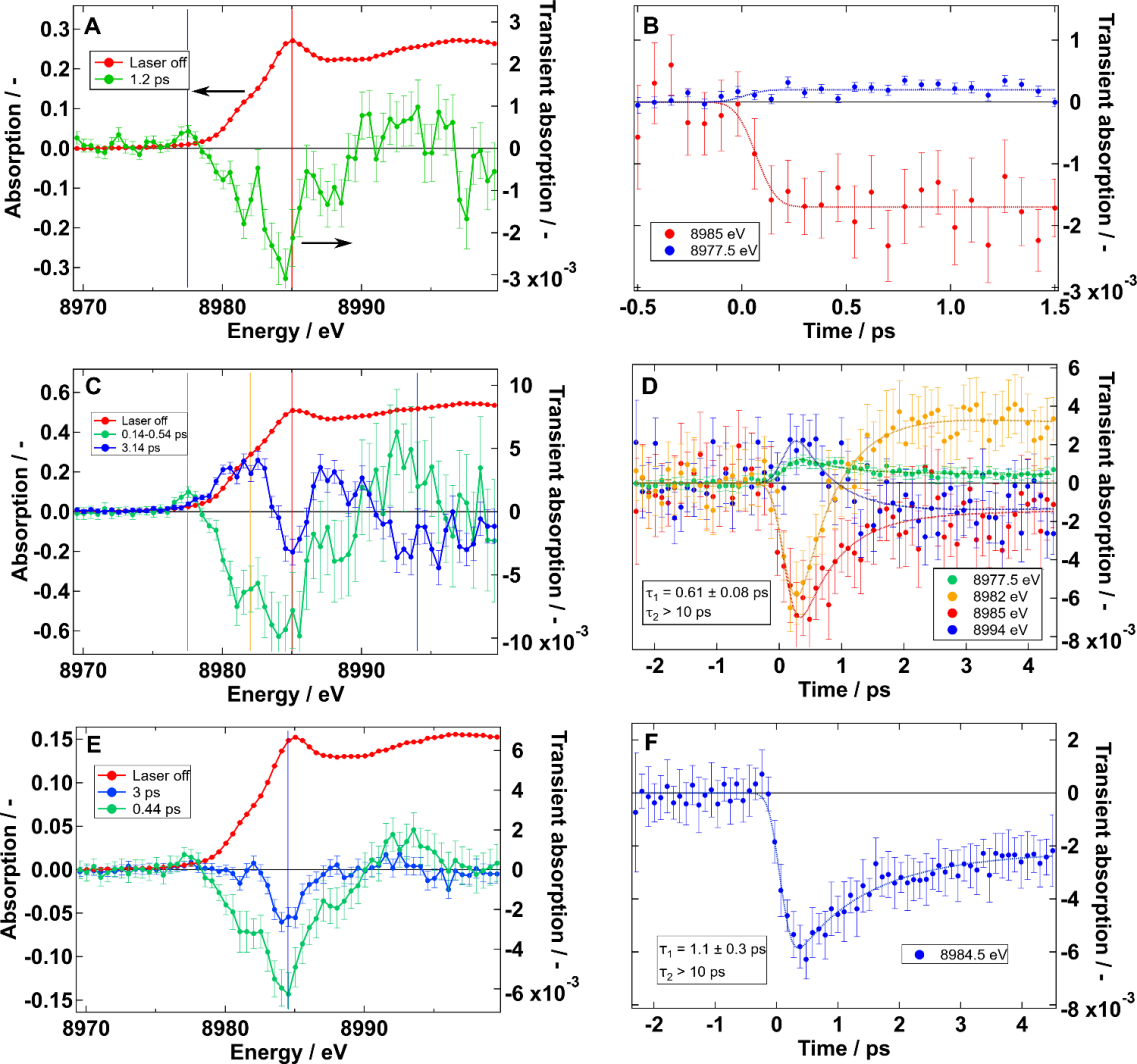
Steady-state (red line) and time-resolved XANES spectra at the Cu K-edge for Cu_0.2_InS_*x*_/ZnS (A), CuIn_0.4_S_*x*_ (C), and Cu_0.3_InS_*x*_ (E). TR-XAS dynamics at specific energies for Cu_0.2_InS_*x*_/ZnS (B), CuIn_0.4_S_*x*_ (D), and Cu_0.3_InS_*x*_ (F). The data were acquired by pumping at 520 nm with fluences of 3 mJ/cm^2^ (A, B), and 13 mJ/cm^2^ (C–F). The error bars correspond to the standard error.

According to our steady-state results, the ground state of CIS QDs is composed of Cu^+^, but excitation at the bandgap of the material photoinduces oxidation from Cu^+^ to Cu^2+^. Therefore, this result agrees with the theoretical calculations showing that the valence band of CIS is mainly composed of Cu atomic orbitals^24^ and with previous time-resolved synchrotron measurements.^29^

Looking at the time-resolved traces in Figure 4B–D, we observe that the dynamics of the Cu_0.2_InS_*x*_/ZnS sample are flat in a time window of 1.5 ps, comparable to what we have observed in the OTAS results. In contrast, the TR-XAS dynamics for the CuIn_0.4_S_*x*_ and the Cu_0.3_InS_*x*_ samples present ultrafast decay processes (Figure 4D,F), due to a hole transfer to trap states unrelated to Cu (**2**). Moreover, for CuIn_0.4_S_*x*_, in less than 3 ps, the Cu^2+^ suffers a reduction that not only brings it back to the initial Cu^+^ state, but it is also mixed with what can be interpreted as structural rearrangement. The former is seen as a disappearance of the large negative band, while the latter is interpreted from the persistent GSB of the 8985 eV peak (1s → 4p) and the appearance of positive side bands. This is explained as an increase in disorder due to thermal effects, producing a broadening.^48^ We can reproduce the same transient by subtracting the steady-state signal of Cu_0.3_InS_*x*_ to the one of CuIn_0.4_S_*x*_ (Figure S11A), which means that the thermal energy of photoexcitation is augmenting the existing difference in crystallinity.

In summary, we observe a clear hole transfer away from Cu sites in both CuIn_0.4_S_*x*_ and Cu_0.3_InS_*x*_ samples. This confirms that such processes are unrelated to Cu states. However, for CuIn_0.4_S_*x*_ this comes together with an increased disorder, perhaps due to partial melting of the quantum dots. Meanwhile, Cu_0.3_InS_*x*_ also shows the same delayed bleach of the 8985 eV peak (1s → 4p), but without the broadening due to disorder. Since neither sample is passivated, we are directly observing the hole transfer to a surface defect or a ligand (**2**). This occurs on the same time scales as the first relaxation steps seen in OTAS, which are associated with electrons (**3**), correlating with the heating effects.

Very similar behavior, as we detected for CuIn_0.4_S_*x*_, was observed for CZTS nanoparticles by Rein et al.^40^ at an even higher fluence (40 mJ/cm^2^ against our 13 mJ/cm^2^). The observed phenomenon was also interpreted as an electron transfer to the Cu sites (hole transfer from them) mixed with structural rearrangement, where heating was influencing even more the measured signal. In the end, the delayed bleach of the 1s → 4p peak after hole transfer may originate from 3 different sources: (i) the effect of electronic relaxation depositing thermal energy in the lattice, (ii) the loss of coordination due to the oxidation of ligands, or (iii) the effects of electrons in the conduction band. This last possibility was also pointed out by Rein et al. as the effect of promoting electrons between the antibonding orbitals of different atom pairs influences the structure in accordance with their EXAFS data. Further measurements would be needed to corroborate these hypotheses unambiguously. Alternatively, the fact that we observe ultrafast back-transfer in both nonpassivated samples, while being absent in the passivated one, allows us to unequivocally assign the lack of PL signal in the former ones to sub-ps hole trapping at surface traps (**2**).

We speculate that these are probably related to the dodecane-thiol ligands, which have been observed to act as hole scavengers in other nanoparticles.^49^ Thus, every other signal that is longer lived either in OTAS or TR-XAS should then be assigned to either the electrons in the conduction band, oxidized ligands or to thermal energy, since the valence band holes have been removed.

Clearly, our TR-XAS measurements of Cu_0.2_InS_*x*_/ZnS were limited by a poor signal-to-noise ratio since we performed those measurements at the lowest pump laser fluence at which they could be detected at the SACLA XFEL. Thus, we are unable at this point to assign the few ps lifetime observed in OTAS to hole localization (**1**), as often suggested in the literature,^14,21,27^ since we did not observe a compatible process in the Cu K-edge, even when probing at longer time-scale scans (Figure S12). Nonetheless, we can rule out ultrafast trapping of photogenerated holes that hypothetically prevents Auger recombination processes, thus supporting our deduction from the steady-state XAS data that Cu-deficient QDs do not have a noticeable concentration of native holes in the form of Cu^2+^, which would lead to such effect.^21,28^ Moreover, it is plausible to assume that the localization of the hole is ultrafast and cannot be resolved, resulting in a direct formation of the clear Cu^2+^ signal with no further evolution, as suggested by Berends et al.^17^ Further X-ray experiments with higher sensitivity (at lower pump fluences to diminish the effect of laser-deposited heat inside the QD) must be carried out to confirm this hypothesis. Alternatively, we do prove that strong sub-ps processes in nonpassivated samples are related to trapping and thermalization, since we observe the hole transfer and the heating effects on the structure.

### Fluorescence Up-Conversion Spectroscopy

Lastly, we performed fluorescence up-conversion spectroscopy (FLUPS) measurements of the Cu-deficient samples, as a probe of emissive states (Figure 5 and Table 2). We find that the initial PL signal of Cu_0.3_InS_*x*_ quickly decays with a lifetime of 1.0 ± 0.3 ps. This correlates well with the hole transfer time at the nonpassivated surface (**2**), observed with TR-XAS (0.7 ± 0.2 ps). While both lifetimes are within their uncertainty, the slightly longer one for FLUPS can be explained through a combination of lower time resolution (∼1 ps) and longer cooling time due to the 400 nm pumping. Interestingly, the PL is also described by a second lifetime of 30 ± 50 ps, this time with great uncertainty due to its weak amplitude.

**Figure 5:**
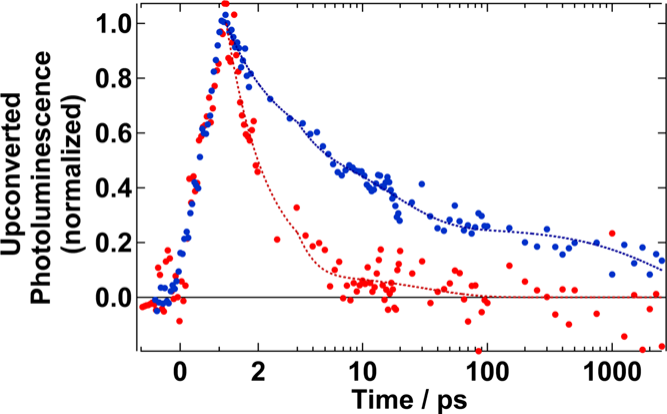
Wavelength-integrated FLUPS signal, for Cu_0.2_InS_*x*_/ZnS (blue) and Cu_0.3_InS_*x*_ (red). Pumping at 400 nm, 3.7 mJ·cm^–2^.

**Table 2:** Photoluminescence Decay Time-Constants Obtained from FLUPS

sample	A_1_/cts	A_2_/cts	A_3_/cts	τ_1_/ps	τ_2_/ps	τ_3_/ps
Cu_0.2_InS_*x*_/ZnS	0.6 ± 0.1 (45%)	0.4 ± 0.1 (30%)	0.33 ± 0.05 (25%)	1.3 ± 0.8	20 ± 10	3 × 10^3^ ± 2 × 10^3^
Cu_0.3_InS_*x*_	0.25 ± 0.3 (93%)	0.02 ± 0.02 (7%)		1.0 ± 0.3	30 ± 50	

This second lifetime can be associated with the possibility of emissive recombination of trapped carriers, albeit with low probability. This can be produced from the trapped states, or through previous detrapping. Due to the weak up-converted signal we cannot distinguish shifts of the PL band that could explain its origin. Alternatively, the good passivation of the Cu_0.2_InS_*x*_/ZnS sample produces a comparatively stronger up-converted signal, with a much longer lifetime. The decrease in τ_1_ and τ_2_ compared to the OTAS results mainly originates from the use of 400 nm excitation. This should be seen as a red-shift of the emission in the first picoseconds due to the cooling to the band edge. However, our setup has a limited spectral region that can be measured. Consequently, the observed shift is very subtle (Figure S16) and we mainly observe this effect as a decay of the signal. This decay can also be produced by a decreased radiative recombination rate due to hole localization (**1**).^27^ Multiexcitonic effects due to the increase in fluence do not affect the fastest dynamics. As seen in Figure S17, an almost 10-fold increase in fluence has small effects.

According to our results, we propose a mechanism for the photophysical processes involved in CIS QDs (Scheme 2). After photoexcitation, an electron is excited from the valence band to the conduction band leaving behind a hole. Since the valence band has a large proportion of Cu atomic orbitals, the hole is observed as an oxidation of Cu. Then, a hole is located at a Cu atom, forming the CHS. This localization appears to be ultrafast, or its effects are not observable with our signal-to-noise. A_1_ in DAS is characterized by changes in the GSB resulting in a red-shift for all samples.

**Scheme 2:**
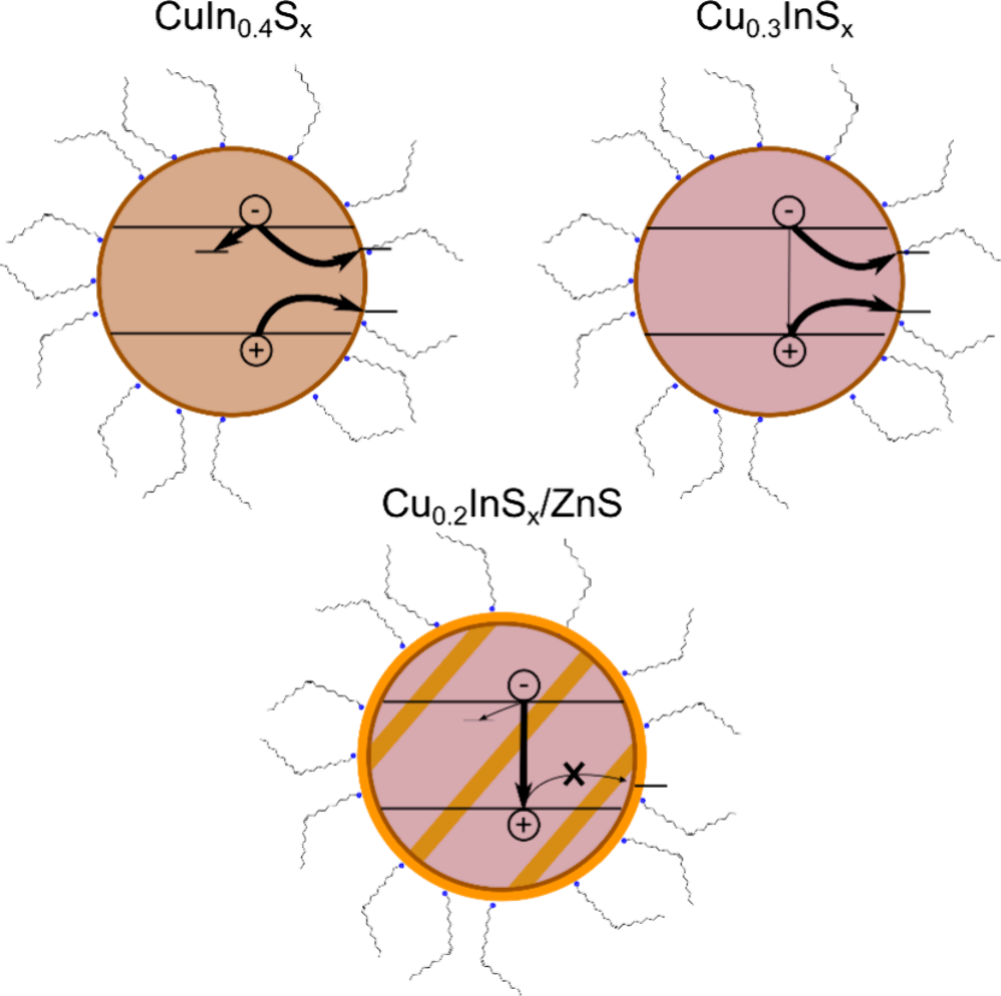
Model Describing the Observed Charge Carrier Dynamics in the Three Different Samples, Ignoring the CHS for Simplicity^a^

a Thick arrows indicate the most probable processes in each system.

This is produced by thermalization to the band edges, including fast trapping of charge carriers for CuIn_0.4_S_*x*_ and Cu_0.3_InS_*x*_ and structural rearrangement of the copper center.

For the nonpassivated samples, three processes occur: thermalization; hole transfer, reducing back the Cu^2+^ to Cu^+^; and electron trapping. For the well-passivated sample, a partial electron trapping may occur which leads to A_2_ in DAS or radiative recombination taking place by recombining the electron delocalized in the conduction band and the hole of Cu^2+^. Due to the 3d^9^ electronic configuration of Cu^2+^, the recombination with the electron in the conduction band is prompt. However, if the Cu^2+^ is quickly reduced to Cu^+^, the PL process will not occur. This explains why the PL efficiency for the Cu-rich sample (CuIn_0.4_S_*x*_), as well as the nonpassivated Cu-deficient one (Cu_0.3_InS_*x*_), is very low compared to the Cu-deficient passivated one (Cu_0.2_InS_*x*_/ZnS). In addition, the disorder produced in CuIn_0.4_S_*x*_ introduces an additional electron trapping process which further quickens charge carrier recombination.

## Conclusions

We have employed a combination of X-ray and optical spectroscopy techniques, both static and time-resolved with femtosecond resolution, to shed new light into the photophysics and structure of CIS QDs. These include highly cutting-edge XFEL measurements with unique observables compared to previous measurements. We have shown that the improvement in PL efficiency with Cu-deficiency originates from a better crystallinity around Cu atoms.

In addition, we have further studied the surface passivation with ZnS. Crystallinity has a stronger effect on electron trapping and thermalization-related structural effects, while surface passivation has a stronger effect on the trapping of both carriers at surface states.

We observed at the Cu K-edge that the Cu-deficient CIS QDs have a more ordered crystal structure than the Cu-rich analogs by looking at the broadening of features in XANES and the decreased coordination in EXAFS. The former was similarly simulated by introducing interstitial defects, while the latter also indicates substantial number of vacancies and some degree of an amorphous phase. The steady-state XANES characterizations were also critical to understand that the ground state of CIS QDs is mainly composed of Cu^+^, independently of the initial concentration of Cu^+^ related to In^3+^. However, this method would not allow us to observe Cu^2+^ concentrations below 20%.

We have further studied our samples through time-resolved techniques probing both at the visible and the hard X-ray range. The latter allowed us to show for the first time the direct observation of hole transfer away from the QD core, with its Cu-dominated valence band, as a quenching mechanism in nonpassivated samples. Furthermore, the hole transfer is produced within the same time scale as electron trapping, with its consequent heat deposition in the lattice and structural rearrangement. This rearrangement increases the structural disorder of the QDs. However, Cu-deficient QDs with their improved crystallinity are resistant to this effect, even at high pump fluence conditions. The second lifetime is related to electron trapping processes in passivated samples, while it is already related to the recombination of trapped carriers in the other two samples. Lastly, Cu_0.2_InS_*x*_/ZnS does not show ultrafast trapping or recombination of holes, as given by the Cu^2+^ photoinduced signal in TR-XAS. This also agrees with a low native Cu^2+^ (hole) concentration, which would lead to Auger recombination.

These results have large implications in the understanding of the structural and compositional effects of synthesis stoichiometry, both contributing to the observed optical properties. Furthermore, they will help the development of CIS QDs and their applications by explaining the reason behind improved optical properties at Cu-deficient synthetic conditions. Moreover, the XFEL results open up the possibility of further element-specific studies on these and other QDs, such as a more systematic study of stoichiometry or to determine the role of different ligands.

## Methods

### CIS/Core Synthesis

First, 0.0587 g of InCl_3_ and 0.0238 g (1:1 Cu:In; 100%) or 0.004 g (0.2:1 Cu:In; 20%) of CuCl were weighed and placed in a 50 mL 3-neck flask. To this flask, 8 mL of 1-octadecene (ODE), 60 μL of oleic acid (OA), and 250 μL of dodecanethiol (DDT) were also added. The mixture was dried under vacuum at 90 °C for 30 min. During this 30 min, a mixture with 0.038 g of sulfur (S) in 3 mL of oleylamine (OAm) was taken to ultrasound for 5 min. After this time, the solution of precursors in ODE was heated to 180 °C under an argon atmosphere for 5 min. Then, the temperature was lowered to 160 °C and 2 mL of the S-OAm solution were injected into the flask, monitoring for 10 min. The solution was cooled in an ice bath to room temperature (25 °C), under stirring and in an inert atmosphere (Argon).

After the synthesis step, the dispersion was transferred to a Falcon centrifuge tube and 8.0 mL of isopropanol were added to purify the NCs. The tube was then taken to the centrifuge for 10 min at 7000 rpm. Finally, the supernatant was removed, and the nanoparticles were suspended in cyclohexane.

This synthesis was adapted from the CZIS core synthesis previously reported,^14^ shown below, removing the Zn precursor.

### CZIS/Core Synthesis

First, 0.0587 g of InCl_3_, 0.0274 g of ZnCl_2_ and 0.004 g (0.2:1 Cu:In) of CuCl were weighed and placed in a 50 mL 3-neck flask. To this flask, 8 mL of 1-octadecene (ODE), 60 μL of oleic acid (OA) and 250 μL of dodecanethiol (DDT) were also added. The mixture was dried under vacuum at 90 °C for 30 min. During this 30 min, a mixture of 0.0257 g of sulfur (S) in 2 mL of oleylamine (OAm) was taken to ultrasound for 5 min. After this time, the solution in the 3-neck flask was heated to 180 °C under an argon atmosphere for 5 min. Then, the temperature was adjusted to 160 °C, and 2 mL of the S-OAm solution was injected into the flask, allowing it to react for 10 min. The solution was cooled in an ice bath to room temperature (25 °C), under stirring and in an inert atmosphere (Argon).

### Synthesis of Zn-OAm Stock Solution

First, 0.2725 g of ZnCl_2_ were weighed and placed in a 50 mL 3-neck flask. To this flask, 4 mL of octadecene (ODE) and 1 mL of oleylamine (OAm) were also added. The mixture was dried under vacuum at 90 °C for 30 min. After this time, the solution was heated at 150 °C under an argon atmosphere for 10 min. Then, the temperature was adjusted to 50 °C.

### CZIS/ZnS Core–Shell Synthesis

The same procedure described above for the CZIS (item 2) was done. With the pristine solution at room temperature and the Zn-OAm stock solution (item 3) at 50 °C, 5 mL of Zn-OAm solution were injected. Then, the system was heated to 200 °C and allowed to react for 30 min. Then, the solution was cooled in an ice bath to room temperature (25 °C), under stirring in an inert atmosphere.

After that, isopropanol was added in a 1:1 ratio to the nanocrystal suspension and centrifuged at 9000 rpm for 10 min. The supernatant was discarded, and the tube remained open for ∼5 min to dry isopropanol residues. The precipitate was suspended in cyclohexane.

### Transient Absorption Spectroscopy

Transient absorption spectroscopy measurements were conducted using a Clark-MXR CPA-1 regenerative amplifier. The fundamental of the laser (775 nm, 1 kHz, 120 fs, 1 mJ) was divided into two paths. One beam supplied a noncollinear optical parametric amplifier (NOPA) to generate 520 nm pulses and filtered to the desired fluence to pump the sample.

The second beam was sent through a CaF_2_ crystal to generate a broadband supercontinuum by self-phase modulation spanning between 380 and 720 nm which was used as the probe. Due to technical circumstances, the Cu_0.3_InS_*x*_ sample had to be probed with a supercontinuum generated with a sapphire crystal, limiting its bandwidth to 480–700 nm. A delay line was used to control the temporal delay between both pulses, which spatially overlapped on the sample. The probe pulse was divided before the sample position between reference and signal beams. The latter was sent through the sample, and both were collected into a prism spectrometer (Entwicklungsburo Stresing GmbH) with a double CCD array. A homemade software recorded the normalized change in absorption (Δ*A*) in a shot-to-shot configuration. All measurements were performed at magic angle (54.7°) between the pump and the probe to avoid anisotropy effects. The samples were measured in 2 mm thin quartz cuvettes with constant stirring with a magnetic bar perpendicular to the incident beam.

### Steady-State X-ray Absorption Spectroscopy

The steady-state XAS spectra were obtained at the CLÆSS beamline of the ALBA synchrotron in Barcelona (Spain).^50^ The X-ray beam was obtained from a multipole wiggler, and monochromatized with a double-crystal monochromator employing Si(111) crystals. The beam was focused down to a spot of 200 × 50 μm^2^ at the sample position. The samples were contained in closed liquid cells with Kapton windows, and the absorption was measured in total fluorescent yield mode. The result was checked for self-absorption by comparing it with the steady-state spectra obtained at the SACLA XFEL, with a 100 μm jet, obtaining comparable spectra.

### Time-Resolved X-ray Absorption Spectroscopy

The TR-XAS experiments were carried out at the X-ray free electron laser facility SPring-8 Angstrom Compact free-electron Laser (SACLA) in Japan, at the BL3 beamline.^51,52^ The X-ray beam was set up with a central energy of 9 keV for Cu K-edge XANES, 500 μJ per pulse and a repetition rate of 30 Hz. At the sample position the X-rays were focused down to a spot of 2 μm in diameter. For excitation, a central wavelength of 520 nm was used, with a spot size of 1040 × 370 μm. The employed pulse energies ranged from 10 to 65 μJ, for fluences of 3 to 21 mJ cm^–2^. The low repetition rate of the SACLA XFEL poses a serious limitation on the signal-to-noise ratio of the recorded TR-XAS signal, leading finally to the necessity of using rather elevated optical pump laser fluences as described in the main text. We have confirmed experimentally the linearity of the detected TR-XAS signal at 13 mJ/cm^2^ (Figure S13) at the shortest time delays and used it throughout the present study. The synchronization system between pump and probe resulted in a jitter that fluctuated between 300 and 1200 fs. This was corrected on the presented data by using a timing tool.^53^ The sample X-ray absorption was measured in a 100 μm cylindrical jet by capturing the X-ray fluorescence yield with a photodiode.

### Fluorescence Up-Conversion Spectroscopy

FLUPS measurements were carried out with a commercial fluorescence up-conversion spectrometer (HALCYONE, Ultrafast Systems). The fundamental output (100 fs and 1 mJ per pulse, λ = 800 nm, 1 kHz repetition rate) of a CPA Ti:sapphire laser (Spitfire, Spectra-Physics) was split into two beams. The first was attenuated and used as a gate. The beam went through a delay stage allowing us to resolve the fluorescence on a 3 ns time window. Then, it was focused onto a rotating BBO crystal for the up-conversion process. The second beam was sent through another BBO crystal for second harmonic generation (400 nm) and used as a pump. The beam was attenuated to the desired excitation pulse energy with a variable ND filter and set to magic angle polarization (54.7°) with respect to the gate using a half-wave plate. Then, the pump beam was sent onto the samples in a transmission geometry and a spot of 49 μm in diameter. The fluorescence was collected and focused into the up-conversion BBO crystal with parabolic mirrors. The up-converted beam was then focused and collected with an optical fiber, sent into a spectrograph, and the spectrum was measured with a thermoelectrically cooled CCD camera.

### Simulations

Calculations of the edge region of the spectrum were performed using the finite difference method as implemented within the FDMNES package.^54^ Throughout, we used a free form SCF potential of radius 6.0 Å around the absorbing atom. Broadening contributions due to the finite mean-free path of the photoelectron and to the core-hole lifetime were accounted for using an arctangent convolution.^55^ Throughout, the crystal structures from Materials Project were used and adjusted as described in the main text.

## Supplementary Material



## Data Availability

The data that support the findings in this study are openly available at the IMDEA Nanoscience repository, ref 56.

## Notes

Nanoparticle characterization with XRF, XRD, and TEM; additional PL characterization and PLQY; analysis of XANES and EXAFS data; additional OTAS spectra and fluence dependence; analysis of TR-XAS spectra and supplementary plots; TRPL results; analysis of FLUPS data with complementary plots; and further information on fitting models (PDF)
