# Supplementary material for: Selective Tracking of Charge Carrier Dynamics in CuInS2 Quantum Dots
Source: ACS Nano. 2025 Jun 12;19(24):21950–61. doi: 10.1021/acsnano.4c18469 (PMC12203636; doi:10.1021/acsnano.4c18469)
Supplement: Supplementary file 1 [file nn4c18469_si_001.pdf]

Supplementary Information

Selective tracking of charge carrier dynamics in $CuInS_2$ quantum dots

Andrés Burgos-Caminal,[*1,2] Brener R. C. Vale,[3,4] André F. V. Fonseca,[5,4] Elisa P. P. Collet,[1,2] Juan F. Hidalgo,[1] Lázaro García,[2] Luke Watson,[6] Olivia Borrell-Grueiro,[7] María E. Corrales,[2,8] Tae-Kyu Choi,[9] Tetsuo Katayama,[10] Dongxiao Fan,[11] Víctor Vega-Mayoral,[1] Saül Garcia-Orrit,[1] Shunsuke Nozawa,[11] Thomas J. Penfold,[6] Juan Cabanillas-González,[1] Shin-Ichi Adachi,[11] Luis Bañares,[7,1] Ana Flávia Nogueira,[5] Lázaro A. Padilha,[3] Marco Antônio Schiavon,[4] and Wojciech Gawelda.[*2,1,12]

1. Madrid Institute for Advanced Studies in Nanoscience, IMDEA Nanociencia, Ciudad Universitaria de Cantoblanco, Calle Faraday 9, 28049 Madrid, Spain.
2. Departamento de Química, Universidad Autónoma de Madrid, Ciudad Universitaria de Cantoblanco, Calle Francisco Tomás y Valiente 7, 28049 Madrid, Spain.
3. Instituto de Física Gleb Wataghin, Universidade Estadual de Campinas - UNICAMP, 13083-852 Campinas, São Paulo, Brazil.
4. Grupo de Pesquisa Química de Materiais, Departamento de Ciências Naturais, Universidade Federal de São João Del-Rei, 36307-352 São João Del-Rei, Minas Gerais, Brazil.
5. Laboratório de Nanotecnologia e Energia Solar, Instituto de Química, Universidade Estadual de Campinas – UNICAMP, 13083-852 Campinas, São Paulo, Brazil.
6. Chemistry-School of Natural and Environmental Sciences, Newcastle University, Newcastle upon Tyne, NE1 7RU, UK.
7. Departamento de Química Física and Center for Ultrafast Lasers, Facultad de Ciencias Químicas, Universidad Complutense de Madrid, 28040 Madrid, Spain
8. Departamento de Química Física Aplicada, Universidad Autónoma de Madrid, Ciudad Universitaria de Cantoblanco, Calle Francisco Tomás y Valiente 7, 28049 Madrid, Spain.
9. XFEL Division, Pohang Accelerator Laboratory, POSTECH, Pohang, Gyeongbuk 37673, Republic of Korea.
10. Japan Synchrotron Radiation Research Institute, Kouto 1-1-1, Sayo, Hyogo 679-5198, Japan.
11. Institute of Materials Structure Science, High Energy Accelerator Research Organization (KEK), 1-1 Oho, 305-0801 Tsukuba, Ibaraki, Japan.
12. Faculty of Physics, Adam Mickiewicz University, ul. Uniwersytetu Poznańskiego 2, 61-614 Poznań, Poland

Corresponding authors: wojciech.gawelda@uam.es, andres.burgos@imdea.org

1. Nanoparticle characterization

To characterize the morphology of the QDs we carried out three different measurements:

- Elemental analysis with X-ray fluorescence (XRF). An S2 PICOFOX from Bruker was used to obtain the relative mass concentration of each element.
- X-ray diffraction (XRD) of the concentrated samples deposited onto a glass slide. The measurements were carried out with a commercial Rigaku SmartLab SE multipurpose X-ray diffractometer. Cu Kα (1.54 Å) was used as the source, and a Bragg-Bentano geometry was used.
- High resolution transmission electron microscopy (TEM) of the washed samples, using a JEM2100 TEM from JEOL.

Table S1. Elemental analysis of the Cu:In ratio carried out with X-ray fluorescence.

	XRF ratio	Mean XRF ratio	Formula
**A**	2.42<br>2.39<br>2.44	2.41 ± 0.03	$CuIn_{0.4}S_x$
**B**	0.305<br>0.254<br>0.275	0.28 ± 0.03	$Cu_{0.3}InS_x$
**C**	0.241<br>0.245<br>0.250	0.245 ± 0.005	$Cu_{0.2}InS_x/ZnS$

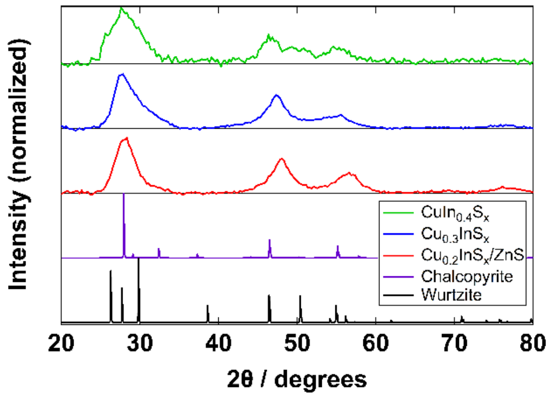

Figure S1. XRD patterns of the three samples. Chalcopyrite[1] and wurtzite[2] patterns simulated from literature crystallographic data with VESTA.[3]

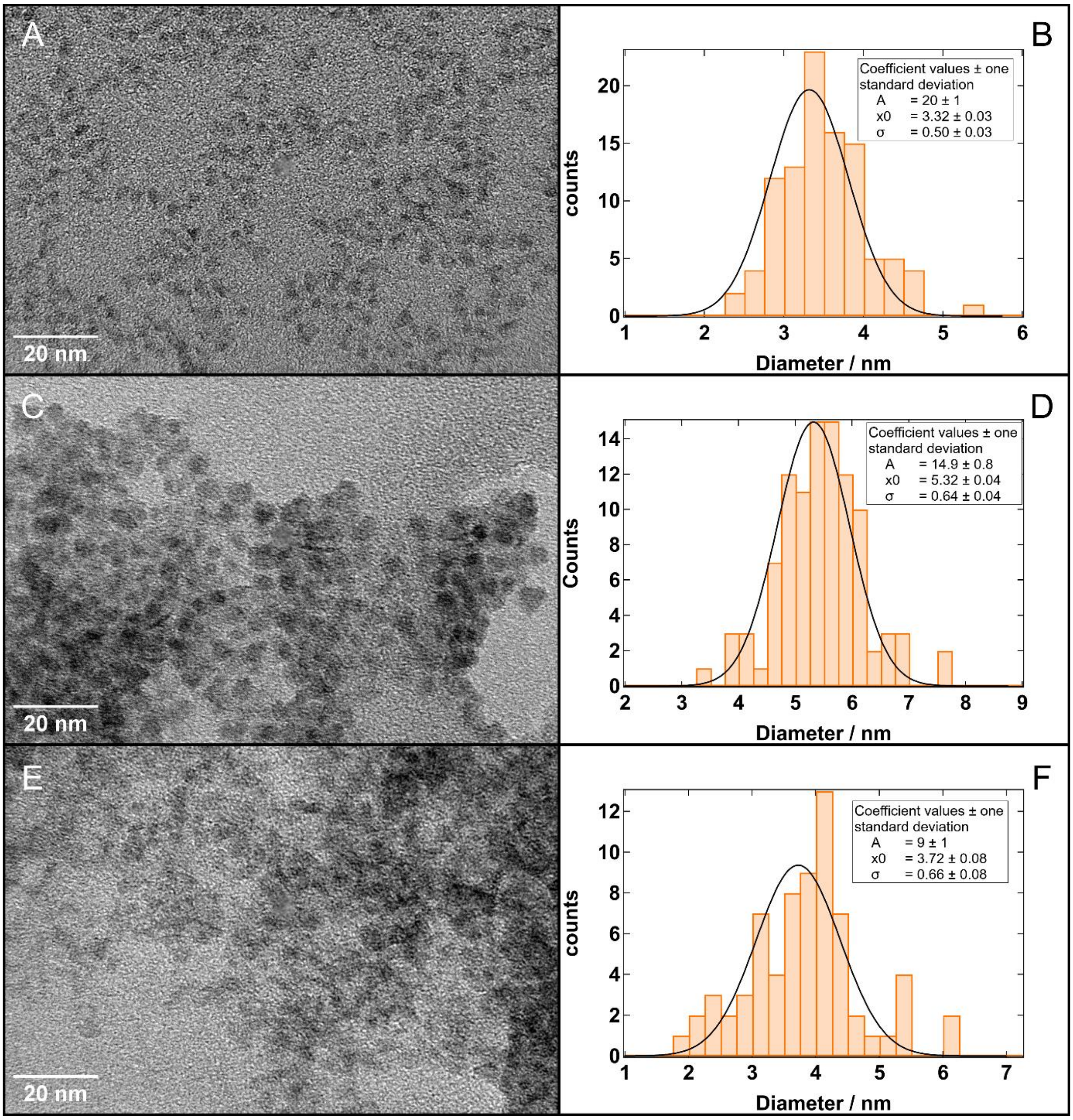

Figure S2. HR-TEM images and the corresponding histograms of $Cu_{0.2}InS_x/ZnS$ (A, B), $CuIn_{0.4}S_x$ (C, D), and $Cu_{0.3}InS_x$ (E, F).

2. Steady-state photoluminescence measurements

Steady-state photoluminescence (PL) measurements were recorded at IMDEA Nanociencia using a Horiba Fluorolog-3 fluorometer. The samples were measured in diluted conditions and the PL was captured at a perpendicular geometry. The samples were excited using 450 nm monochromatized light from a light bulb.

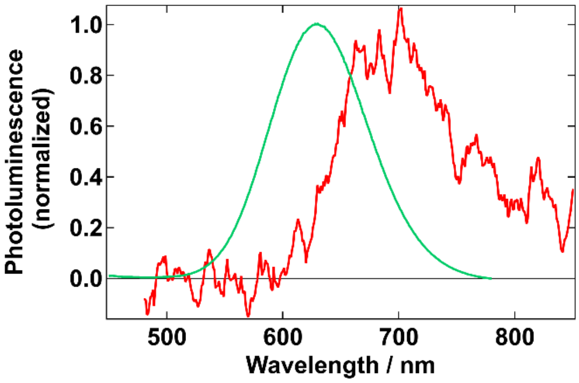

Figure S3. Normalized PL of $Cu_{0.2}InS_x/ZnS$ (green), and $Cu_{0.3}InS_x$ (red) Only $Cu_{0.2}InS_x/ZnS$ is clearly emissive. $Cu_{0.3}InS_x$ shows a very weak band, while $CuIn_{0.4}S_x$ does not show any band above measurement artifacts and noise, and it is thus excluded.

Photoluminescence quantum yield (PLQY) measurements were carried out with a Quantaurus Plus, Hamamatsu, Pl quantum yield spectrometer. The statistics were obtained from different measurements at different wavelengths (Table S1). The results do not vary as a function of wavelength.

Table S2. PLQY results.

**λ / nm**	400	410	420	430	440	450	460	470	480	490
**PLQY / %**	29.5	30.1	29.1	29.7	29.6	30.0	29.8	30.4	28.6	31.6

3. Analysis of steady-state XANES and EXAFS data (CLAESS beamline, ALBA)

To be able to compare with our samples presented in the article (Fig. 2) we measured two references, Cu_2S and CuS, at ALBA synchrotron. These are examples of Cu^+ and Cu^{2+}, respectively. Sulfides were chosen as closer references to $CuInS_2$.

Unlike the QD samples presented in the main text, which were measured as dilutions and in total fluorescence yield mode, these references were measured in the form of pellets dispersed in cellulose and in transmission mode.

All the data was treated with the Athena program,[4] correcting the background and normalizing to the EXAFS region.

On Fig. S2 we analyze these reference spectra. On the left we can appreciate the small band characteristic of Cu^{2+} at 8979 eV due to the $1s^2 \rightarrow 3d^9$ transition. This is easier to notice in the derivative spectrum (right) as shown by the arrow in the blue curve. However, our reference measurements present rather broad features, and substantial noise, unlike our sharper sample measurements in Fig. 2. This is a result of measuring the references in the form of pellets, in contrast to the sample liquid colloidal suspensions. Better examples can be found in the literature.[5,6]

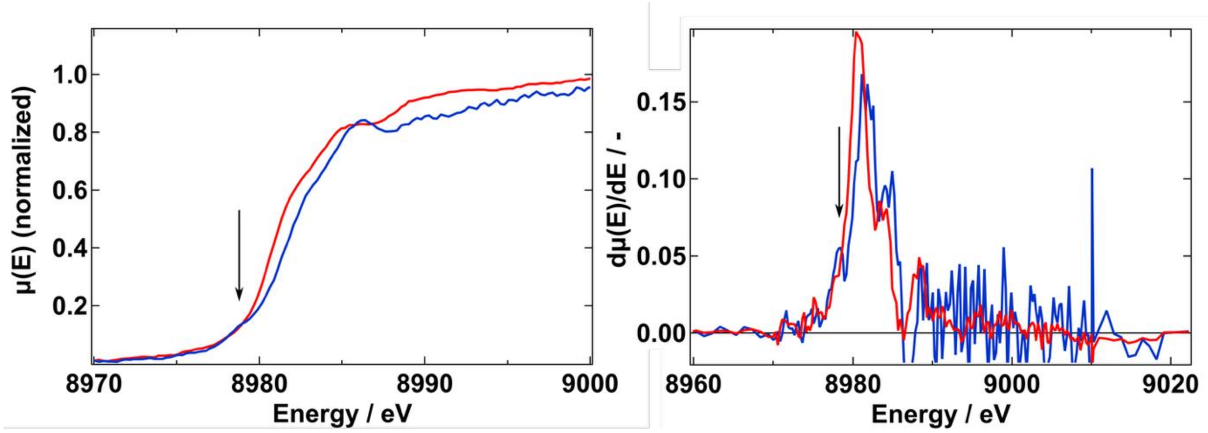

Figure S4. Reference XANES spectra (CuS, blue, and Cu_2S, red) at the Cu K-edge. Notice the signature of $1s^2 \rightarrow 3d^9$ transition, as well as the general chemical shift of all features.

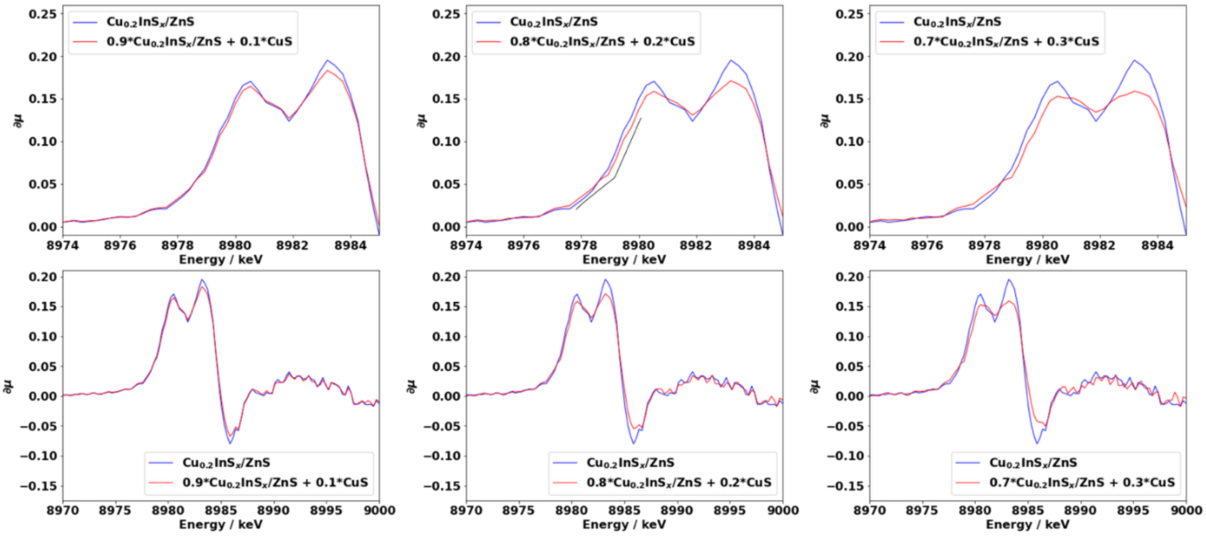

Figure S5. Derivative spectra of $Cu_{0.2}InS_x/ZnS$ and a combination of $Cu_{0.2}InS_x/ZnS$ with a 10, 20 and 30% of CuS to distinguish the effect of Cu^{2+}. Both the full spectrum (bottom row) and a zoom in around the $1s^2 \rightarrow 3d^9$ transition area (top row) are shown. Notice the clear change in slope for a 20% contribution of Cu^{2+}, in addition to the broadening of the peaks.

When compared with the XANES spectrum of $Cu_{0.2}InS_x/ZnS$ in Fig. S3, all the pre-edge features align with the Cu^+ reference (Cu_2S).

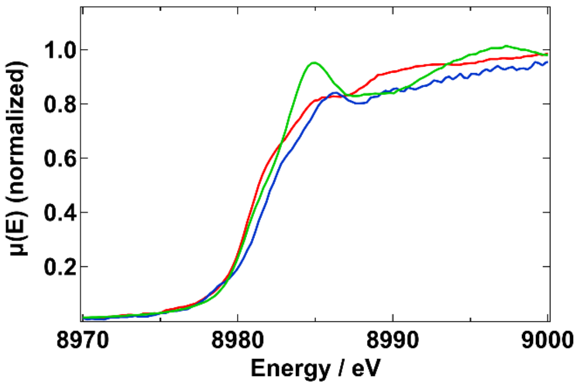

Figure S6. Comparison between the XANES spectra of the two references (CuS, blue, and Cu_2S, red) and $Cu_{0.2}InS_x/ZnS$ (green).

EXAFS analysis was carried out with the Artemis program,[4] applying the in-built FEFF6 for the fit. All samples were fitted in a similar way.

k-range = 2.1 - 13

k-weight = 1,2

R-range = 1.35 – 5

The background removal was carried out with a k-weight of 2, an Rbkg of 1.3 and an E0 of 8983.3 eV.

The crystal structure of $CuInS_2$ was obtained from a reference and used for the FEFF calculation.[1] The resulting direct scattering paths with S (1st shell), Cu and In (2nd shell) were used to fit the EXAFS equation:[7]

$$\chi(k) = \sum_j \frac{S_0^2 N_j f_j(k) e^{-2R_j/\lambda(k)} e^{-2k^2\sigma_j^2}}{kR_j^2} \sin[2kR_j + \delta_j(k)], \quad (S1)$$

Where we are interested in fitting N_j, the coordination number, R_j, the near neighbor distance, and σ_j, the mean square disorder. S_0^2 can be obtained from literature or from the fit of a well-known reference.

We obtained reasonable fits with R-factors of 0.0135, 0.0156 and 0.0232 for $CuIn_{0.4}S_x$, $Cu_{0.3}InS_x$ and $Cu_{0.2}InS_x/ZnS$, respectively.

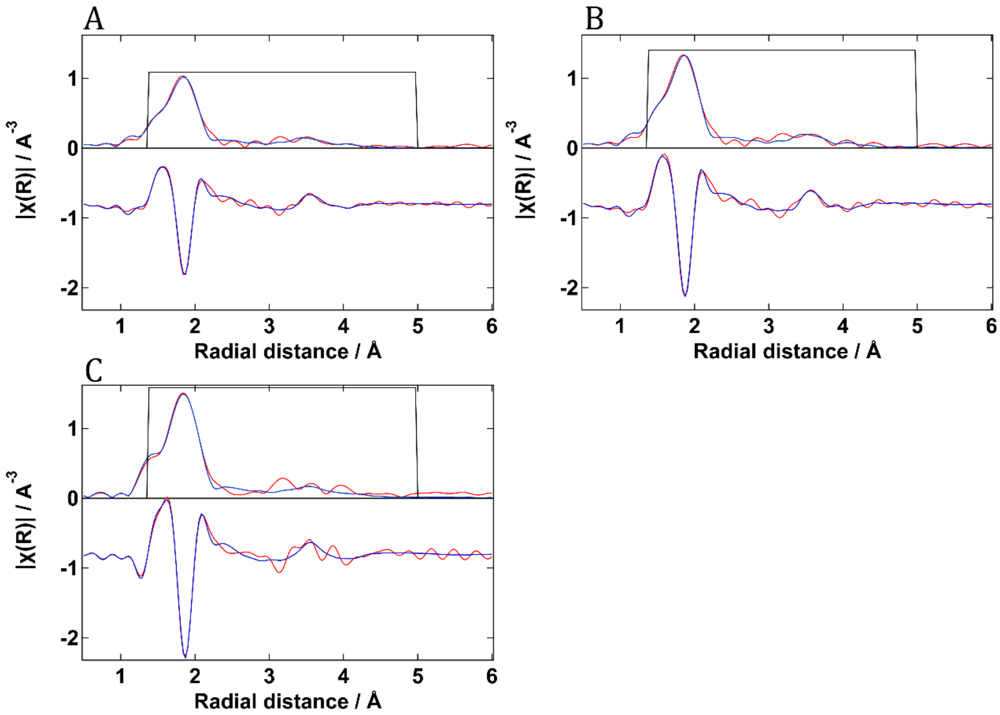

Figure S7. FEFF6 fits of $CuIn_{0.4}S_x$ (A), $Cu_{0.3}InS_x$ (B), and $Cu_{0.2}InS_x$/ZnS (C).

Table S3: Cu K-edge EXAFS FEFF fitting parameters for the single scattering path with the first shell of S atoms. We assume an S_0^2 of 0.9 as a reasonable estimate, obtained from an EXAFS fit of metallic Cu.

Sample	$N{\cdot}S_0^2$	N	σ^2	R
**$CuIn_{0.4}S_x$**	2.1 ± 0.1	2.3 ± 0.1	0.0076 ± 0.0009	2.301 ± 0.005
**$Cu_{0.3}InS_x$**	2.7 ± 0.2	3.0 ± 0.2	0.008 ± 0.001	2.316 ± 0.005
**$Cu_{0.2}InS_x$/ZnS**	3.0 ± 0.3	3.4 ± 0.3	0.007 ± 0.001	2.314 ± 0.007

4. Time-resolved optical TAS data

In Fig. 2 we show the result of a global fit on the OTAS data. More details are given in section 8. Instead, here in Fig. S5 we show the ΔA spectra at given times.

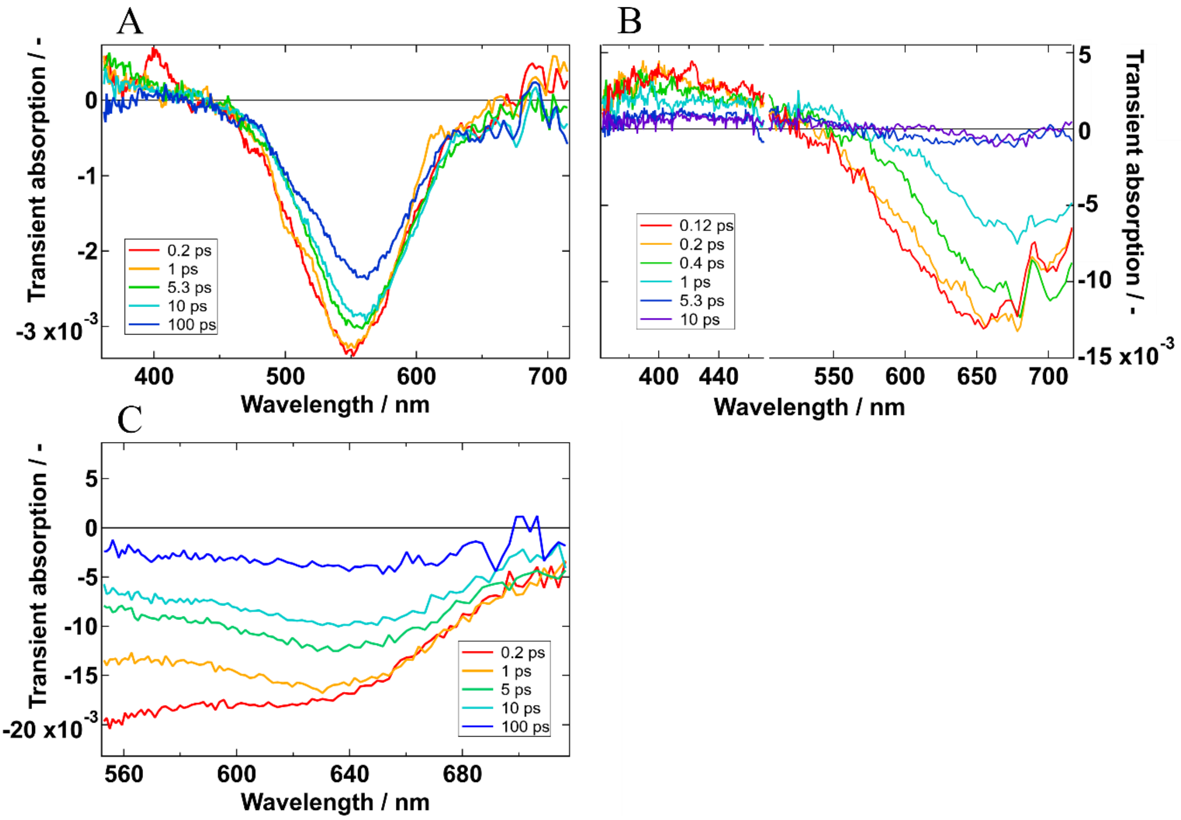

Figure S8. Transient absorption spectra at different time delays of $Cu_{0.2}InS_x$/ZnS (A), $CuIn_{0.4}S_x$ (B), and $Cu_{0.3}InS_x$ (C).

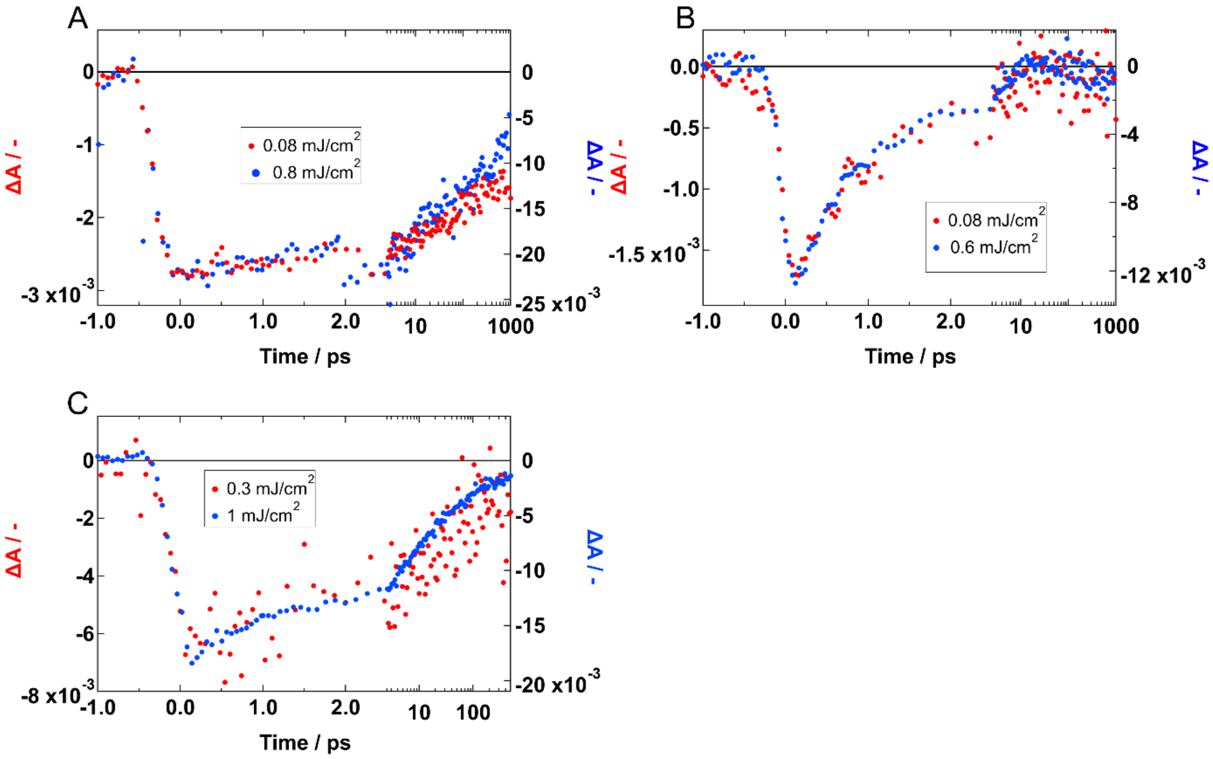

Figure S9. Fluence dependent TAS measurements of A) $Cu_{0.2}InS_x/ZnS$ (550 nm), B) $CuIn_{0.4}S_x$ (650 nm), and C) $Cu_{0.3}InS_x$ (590 nm). The main effect of the fluence lies in the tens and hundreds of picoseconds, mainly seen for $Cu_{0.2}InS_x/ZnS$ where there is no fast trapping.

5. Analysis of time-resolved XANES data (SACLA XFEL)

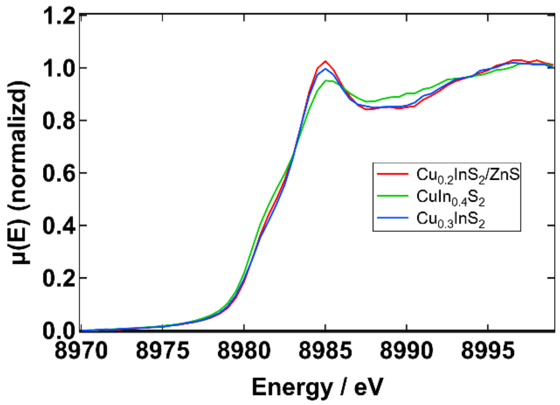

Figure S10. XANES spectra of the three samples under study measured at the SACLA XFEL facility.

To further illustrate the processes assigned to the transients of our TR-XAS data, we have simulated three transients.

First, the difference between the XAS spectra of $CuIn_{0.4}S_x$ and $Cu_{0.3}InS_x$, to show that the increase in disorder between these two samples is very similar to the obtained transient in the former.

Second, the effect of a general broadening due to disorder. This is obtained by convoluting the XAS spectrum of $CuIn_{0.4}S_x$ with a Gaussian profile. The result agrees very well with the long-lived transient spectrum of $CuIn_{0.4}S_x$. However, the transient lacks the pronounced peak at 8979.5 eV. This is because in the data the broadening affects mainly the $1s^2 \rightarrow 4p$ peak at 8985.

Third, the effect of a general blue-shift of the spectrum of $CuIn_{0.4}S_x$ due to oxidation. The broad negative feature resembles the early times transient. However, it lacks the appearance of the $1s^2 \rightarrow 3d^9$ peak at 8977.5 eV due to the formation of Cu^{2+}, present in the experimental data.

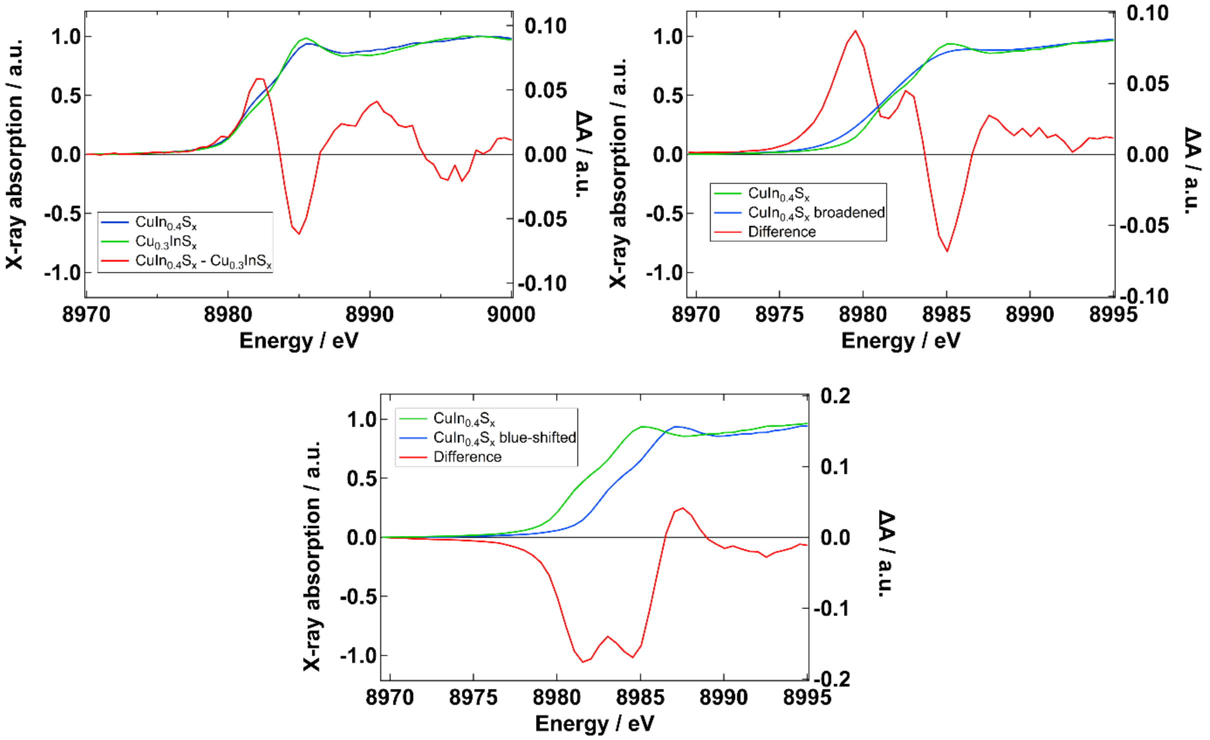

Figure S11. A) Difference XAS between $CuIn_{0.4}S_x$ and $Cu_{0.3}InS_x$ showing a very similar change to the long-lived transient of $CuIn_{0.4}S_x$. B) Simulated effect of a general broadening on the XAS spectrum of $CuIn_{0.4}S_x$. The shape resembles closely the transient at longer times, with an additional band at low energy. C) Simulated effect of a general blue-shift (oxidation) on the XAS spectrum of $CuIn_{0.4}S_x$. The large negative band agrees with the transient spectrum at early times, although it lacks the appearance of the $1s^2 \rightarrow 3d^9$ peak at 8977.5 eV.

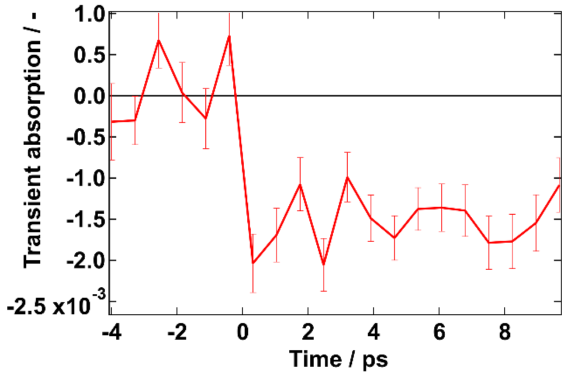

Figure S12. Extended window for the time-resolved XANES of $Cu_{0.2}InS_x$/ZnS at 8985 eV, shown in Figure 4.

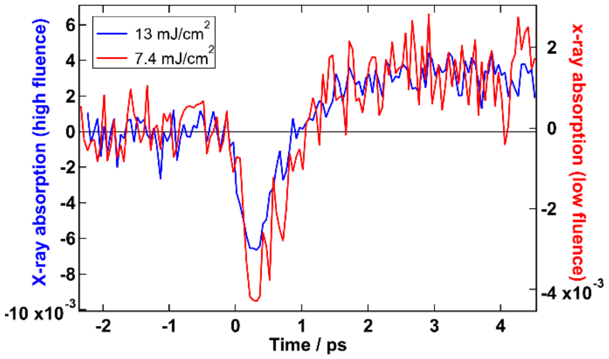

Figure S13. Fluence dependent TR-XAS of $CuIn_{0.4}S_x$ at 8982eV, where the signal scales linearly with the fluence and the decay rates are similar within the noise.

6. Time-resolved photoluminescence measurements

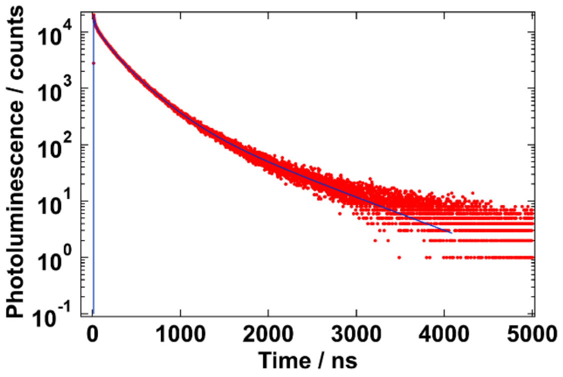

Figure S14. Time-resolved PL of $Cu_{0.2}InS_x/ZnS$ showing a multiexponential decay.

IRF/ns	0.996 ± 0.005
t_0/ns	11.475 ± 0.003
A_1/cts	$4.34 \cdot 10^3 \pm 7 \cdot 10^1$
A_2/cts	$3.91 \cdot 10^3 \pm 6 \cdot 10^1$
A_3/cts	$7.3 \cdot 10^3 \pm 3 \cdot 10^2$
A_4/cts	$5.6 \cdot 10^3 \pm 3 \cdot 10^2$
A_5/cts	$6.5 \cdot 10^2 \pm 9 \cdot 10^1$
τ_1/ns	3.5 ± 0.1
τ_2/ns	19.2 ± 0.5
τ_3/ns	$2.73 \cdot 10^2 \pm 8$
τ_4/ns	$1.2 \cdot 10^2 \pm 4$
τ_5/ns	$7.4 \cdot 10^2 \pm 3 \cdot 10^1$

Table S4. Fit results of the TRPL data in Figure S14 for a 5-exponential model.

We can assume that the last three components will be the ones corresponding to particles free of electron trapping. Then the averaged radiative lifetime is calculated as an intensity-weighted average of the last 3 components as follows:

$$\frac{\sum_n \tau_n^2 A_n}{\sum_n \tau_n A_n} \quad \text{(S2)}$$

Obtaining an average radiative lifetime of 310 ns.

7. Analysis of FLUPS data

The time-resolved detection of the PL through up-conversion depends highly on the PL intensity at any given time. If a material has a very long PL lifetime, it will emit few photons per 100 femtoseconds. Therefore, even if the material has high PL quantum yield (PLQY) it will be hard to detect it with FLUPS. This is the case for CIS QDs, even for $Cu_{0.2}InS_x/ZnS$, hence the use of considerable fluences in the measurements.

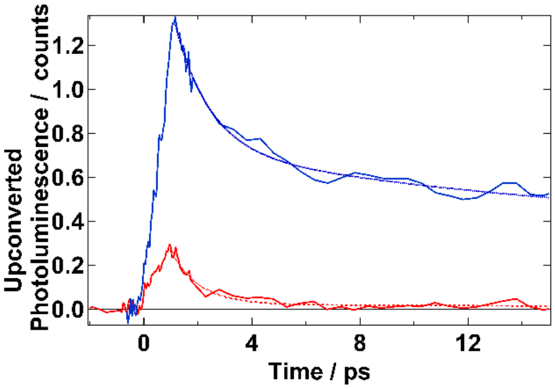

Figure S15. Linear scale and not normalized version of Fig 5, showing $Cu_{0.2}InS_x/ZnS$ (blue) and $Cu_{0.3}InS_x$ (red). The time zero has been shifted in Fig. 5 to accommodate the log scale.

Another source of difficulty in FLUPS is the fact that the response of the setup is not equal at all wavelengths. A photometric correction has been employed in the past to correct this problem.[8] However, our poor spectral resolution compared to a standard UV-vis makes the spectra hardly comparable. Furthermore, with CIS QDs there is a larger issue. To avoid contamination from the 400 nm light employed as pump, a short-pass filter is employed that only lets through the up-converted UV radiation. This translates in an abrupt cut of the PL detection at 650 nm. In conclusion, the obtained spectra are limited, and these limitations should be considered. Here, we show a qualitative comparison in Fig. S16, while throughout the other figures a spectral integration is carried out to maximize the signal of the temporal traces. This seems to distort slightly the rise of the signal. Thus, instead of employing the convoluted exponential explained in the next section, we fitted standard exponential equations to the decays starting at the maximum.

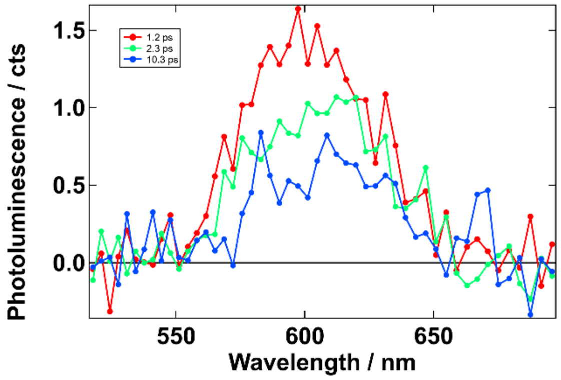

Figure S16. FLUPS spectra of $Cu_{0.2}InS_x/ZnS$ at different time delays. Notice that a shift on the maximum is apparent. The detection efficiency of our setup quickly decays above 600 nm, cutting completely at 650 nm. Consequently, the shift is only observed as a faster decay of the blue side of the band.

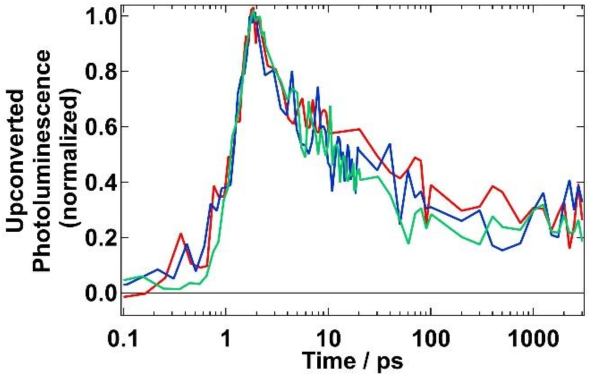

Figure S17. Upconverted photoluminescence of $Cu_{0.2}InS_x/ZnS$ at 0.8 (red), 2.6 (blue), and 5.8 (green) mJ/cm^2.

7. Exponential models

To carry out the fits of the time-resolved dynamics we employed an exponential decay convoluted with a Gaussian resulting in the equation:

$$S(t) = A\frac{1}{2}\exp\left(\frac{4ln(2)w^2}{\tau^2} - \frac{t-t_0}{\tau}\right)\left(1 + \mathrm{erf}\left(\frac{t-t_0}{4\sqrt{ln(2)}\,w} - \frac{2\sqrt{ln(2)}w}{\tau}\right)\right) \quad (S3)$$

Where A is the amplitude, w is the Full width at half maximum, τ is the lifetime, and t0 is the time zero. For a multiexponential decay, we employ the sum of several such equations.

8. Global fit

We carried out global fits for the OTAS and TR-XAS data using the Igor Pro built-in in package. For any global fit, a set of equivalent multiexponential equations (sum of Eq. S3) equal to the number of time traces to be analyzed is fitted simultaneously. Each time trace corresponds to a different wavelength or energy. The parameters w and τ_n are treated as common for all time traces, obtaining best fit values for all of them. On contrary, a different value of A_n parameter is fitted for each time trace. The spectral representation of A_n against wavelength or energy is called decay associated spectrum (DAS), as shown in Fig. 3. The DAS allow us to interpret the spectral changes produced by each exponential decay with their associated τ.

References

(1) Hahn, H.; Frank, G.; Klingler, W.; Meyer, A.-D.; Störger, G. Untersuchungen über ternäre Chalkogenide. V. Über einige ternäre Chalkogenide mit Chalkopyritstruktur. *Z. Für Anorg. Allg. Chem.* **1953**, *271*, 153–170. https://doi.org/10.1002/zaac.19532710307.

(2) Li, Q.; Zhai, L.; Zou, C.; Huang, X.; Zhang, L.; Yang, Y.; Chen, X.; Huang, S. Wurtzite CuInS2 and CuInxGa1−xS2 Nanoribbons: Synthesis, Optical and Photoelectrical Properties. *Nanoscale* **2013**, *5*, 1638–1648. https://doi.org/10.1039/C2NR33173J.

(3) Momma, K.; Izumi, F. Vesta 3 for Three-Dimensional Visualization of Crystal, Volumetric and Morphology Data. *J. Appl. Crystallogr.* **2011**, *44*, 1272–1276. https://doi.org/10.1107/S0021889811038970.

(4) Ravel, B.; Newville, M. ATHENA, ARTEMIS, HEPHAESTUS: Data Analysis for X-Ray Absorption Spectroscopy Using IFEFFIT. *J. Synchrotron Radiat.* **2005**, *12*, 537–541. https://doi.org/10.1107/S0909049505012719.

(5) Alwis, L. K. H. K.; Mucalo, M. R.; Ingham, B.; Kappen, P. A Combined SNIFTIRS and XANES Study of Electrically Polarized Copper Electrodes in DMSO and DMF Solutions of Cyanate (NCO−), Thiocyanate (NCS−) and Selenocyanate (NCSe−) Ions. *J. Electrochem. Soc.* **2015**, *162*, H434. https://doi.org/10.1149/2.0321507jes.

(6) Frenkel, A. I.; Korshin, G. V.; Ankudinov, A. L. XANES Study of Cu2+-Binding Sites in Aquatic Humic Substances. *Environ. Sci. Technol.* **2000**, *34*, 2138–2142. https://doi.org/10.1021/es990561u.

(7) Newville, M. EXAFS Analysis with Feff, Larch, Artemis, 2018. https://millenia.cars.aps.anl.gov/videos/FundamentalsOfXAFS/UsingFeff.pdf.

(8) Zhang, X.-X.; Würth, C.; Zhao, L.; Resch-Genger, U.; Ernsting, N. P.; Sajadi, M. Femtosecond Broadband Fluorescence Upconversion Spectroscopy: Improved Setup and Photometric Correction. *Rev. Sci. Instrum.* **2011**, *82*, 063108. https://doi.org/10.1063/1.3597674.
